# Supplementary material for: Catalytic transformation of dinitrogen into ammonia and hydrazine by iron-dinitrogen complexes bearing pincer ligand
Source: Nat Commun. 2016 Jul 20;7:12181. doi: 10.1038/ncomms12181 (PMC4961768; doi:10.1038/ncomms12181)
Supplement: Supplementary Information — Supplementary Figures 1-11, Supplementary Tables 1-23, Supplementary Methods and Supplementary References [file ncomms12181-s1.pdf]

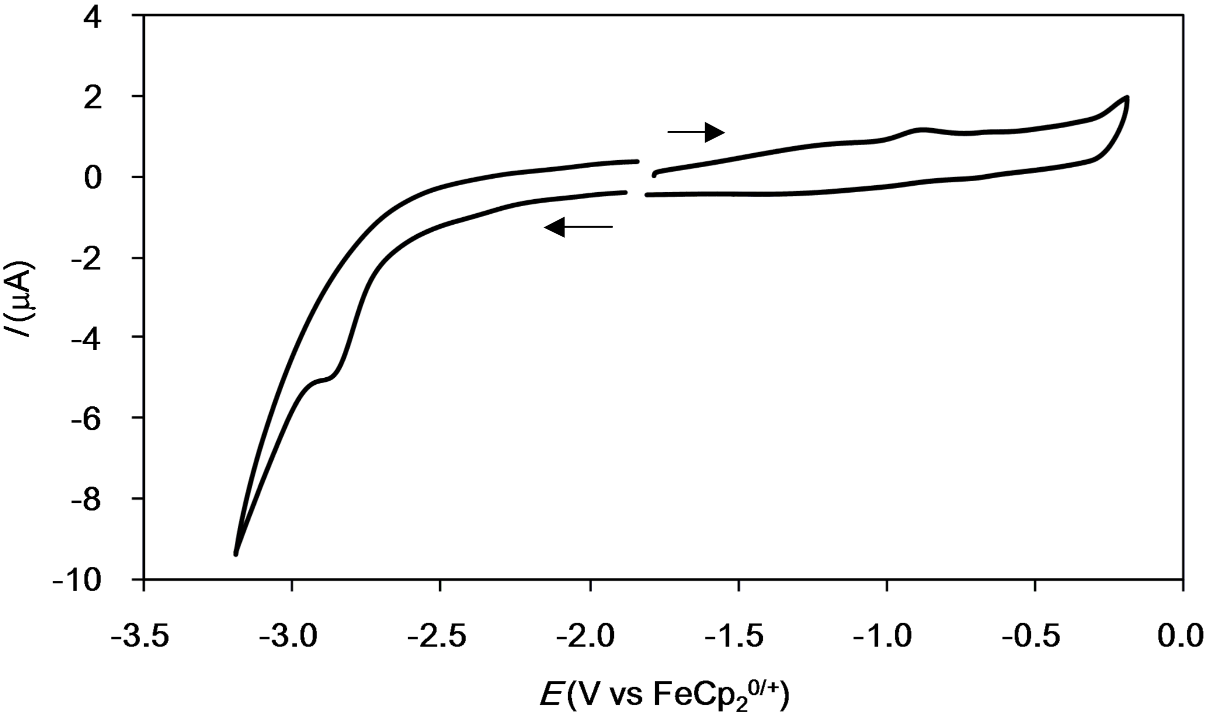

Supplementary Figure 1 | Cyclic voltammograms of the dinitrogen complex 1. The cyclic voltammetry of **1** revealed an irreversible reduction at -2.9 V vs ferrocene$^{0/+}$ and an irreversible oxidation at -0.9 V. According to this result, the energy of the HOMO-LUMO gap was estimated to be *ca.* 2.0 eV.

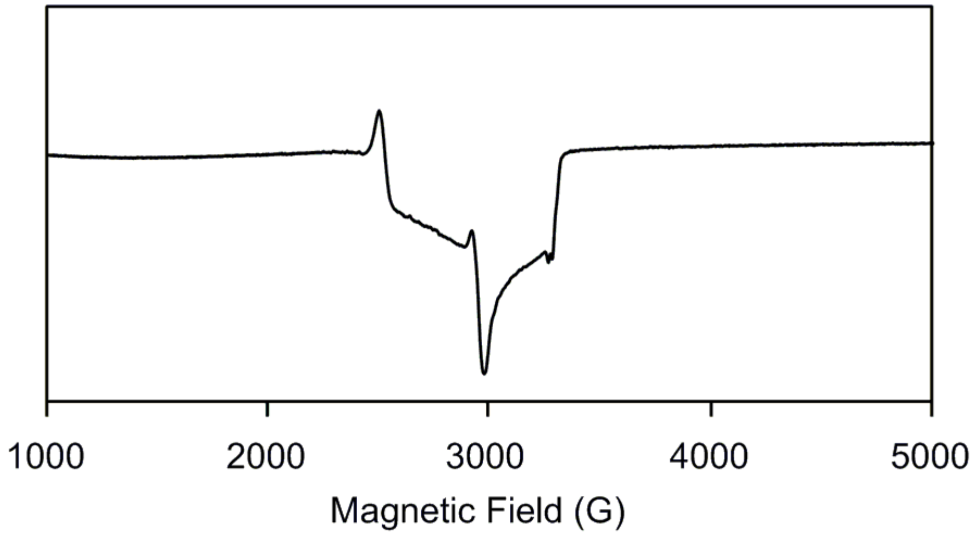

Supplementary Figure 2 | X-band EPR spectra of 1. The spectrum collected at 10 K in a toluene glass at a microwave frequency 9.07 GHz. Reproducible ESR signals at g = 2.6 and 2.2 are attributable to the ESR of **1**, although, a broad signal from 2500 G to 3300 G is considered to be derived from unidentified decomposed species.

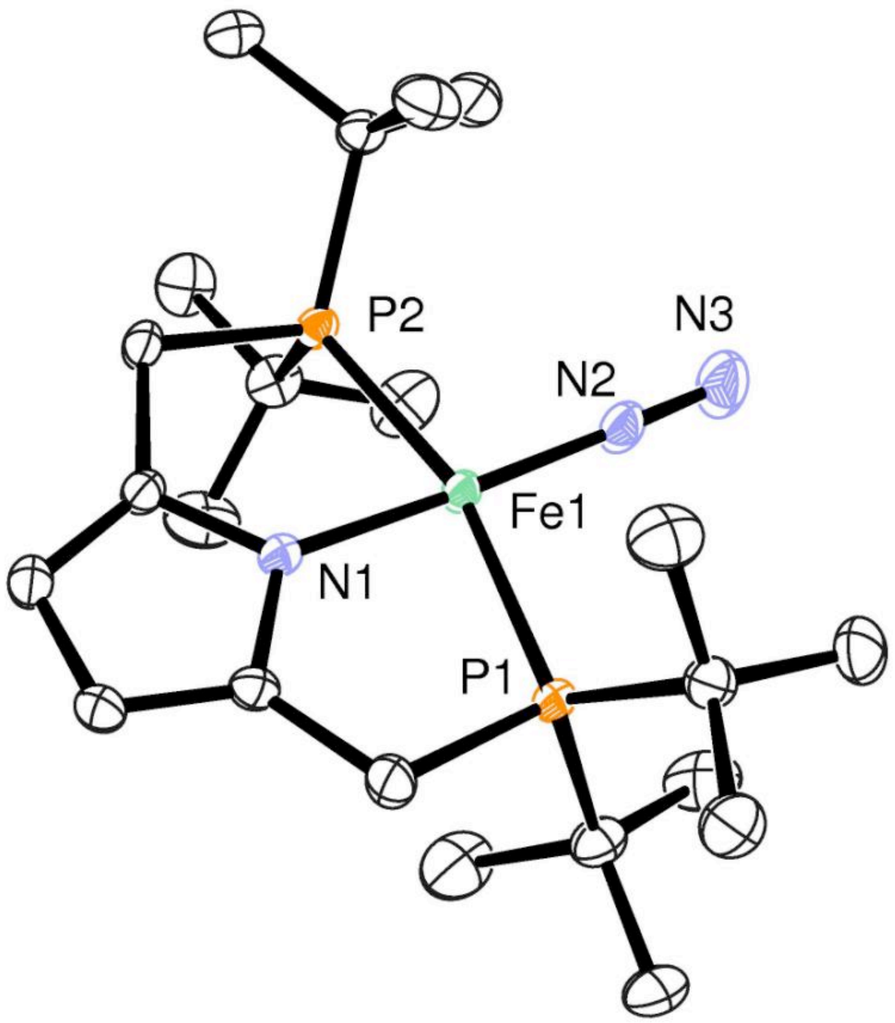

Supplementary Figure 3 | ORTEP drawing of 1. Thermal ellipsoids are shown at the with the 50% probability level. Hydrogen atoms are omitted for clarity. Selected bond lengths (Å) and angles (deg): Fe(1)–N(1) = 1.9144(14), Fe(1)–N(2) = 1.7643(16), Fe(1)–P(1) = 2.2788(4), Fe(1)–P(2) = 2.2740(4), N(2)–N(3) = 1.134(2), N(1)–Fe(1)–N(2) = 176.81(6), P1(1)–Fe(1)–P(2) = 164.158(13), N(1)–Fe(1)–P(1) = 82.89(3), = N(2)–Fe(1)–P(1) = 98.14(4), N(1)–Fe(1)–P(2) = 82.72(3), N(2)–Fe(1)–P(2) = 96.58(4), Fe(1)–N(2)–N(3) = 178.50(14).

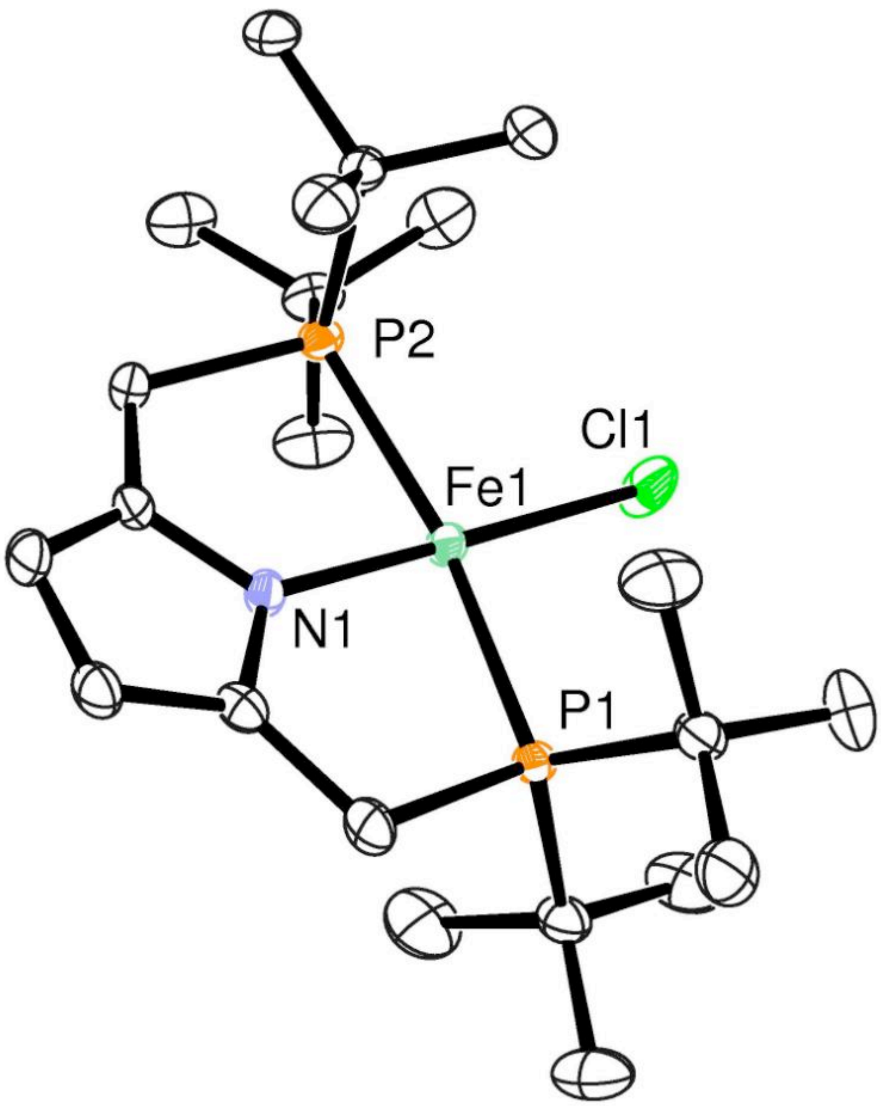

Supplementary Figure 4 | ORTEP drawing of 2. Thermal ellipsoids are shown at the with the 50% probability level. Hydrogen atoms are omitted for clarity. Selected bond lengths (Å) and angles (deg): Fe(1)–N(1) = 1.8901(16), Fe(1)–Cl(1) = 2.2433(7), Fe(1)–P(1) = 2.2952(6), Fe(1)–P(2) = 2.2860(6), N(1)–Fe(1)–Cl(1) = 178.43(5), P1(1)–Fe(1)–P(2) = 165.80(3), N(1)–Fe(1)–P(1) = 83.54(5), Cl(1)–Fe(1)–P(1) = 97.79(3), N(1)–Fe(1)–P(2) = 83.17(5), Cl(1)–Fe(1)–P(2) = 95.58(3).

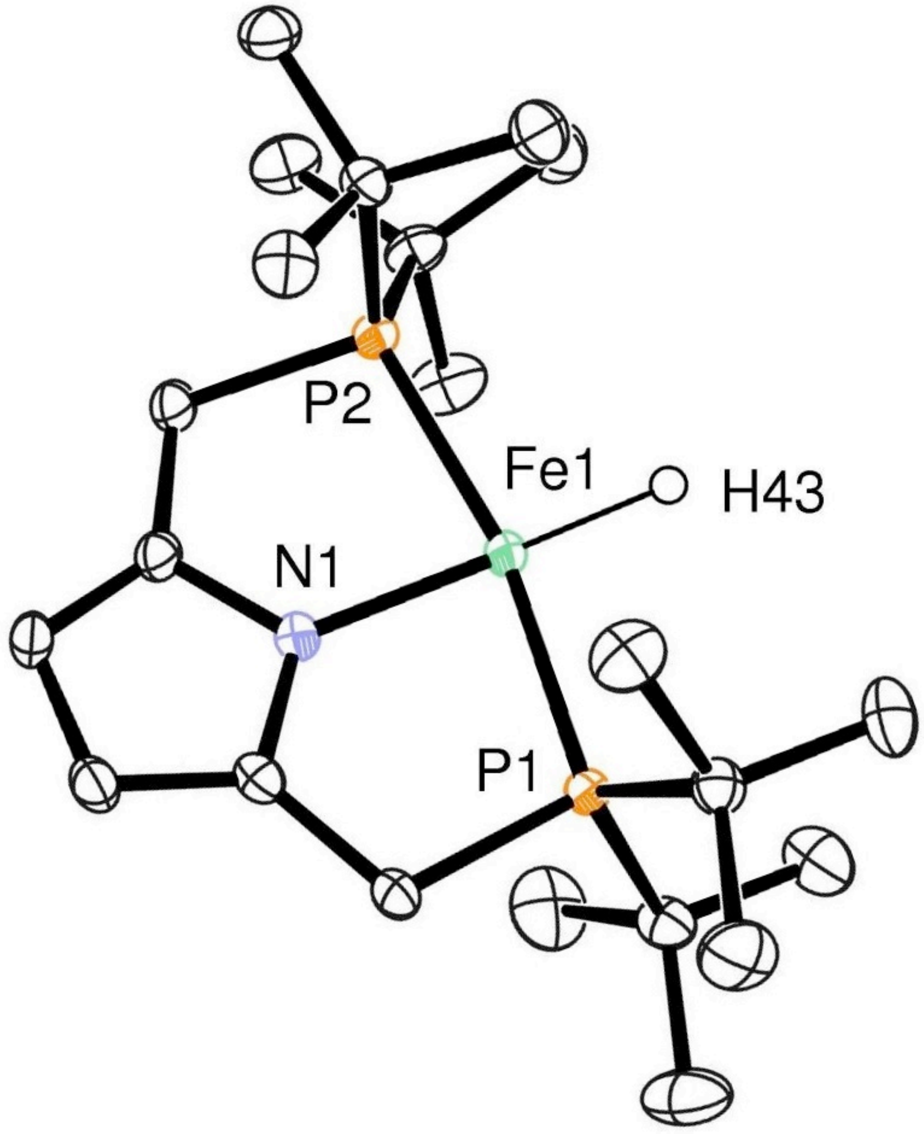

Supplementary Figure 5 | ORTEP drawing of 3. Thermal ellipsoids are shown at the with the 50% probability level. Hydrogen atoms except for H43 are omitted for clarity. Selected bond lengths (Å) and angles (deg): Fe(1)–N(1) = 1.9256(19), Fe(1)–H(43) = 1.58(3), Fe(1)–P(1) = 2.2497(8), Fe(1)–P(2) = 2.2439(8), N(1)–Fe(1)–H(43) = 178.1(10), P1(1)–Fe(1)–P(2) = 166.46(3), N(1)–Fe(1)–P(1) = 83.33(6), H(43)–Fe(1)–P(1) = 95.2(10), N(1)–Fe(1)–P(2) = 84.15(6), H(43)–Fe(1)–P(2) = 97.4(10).

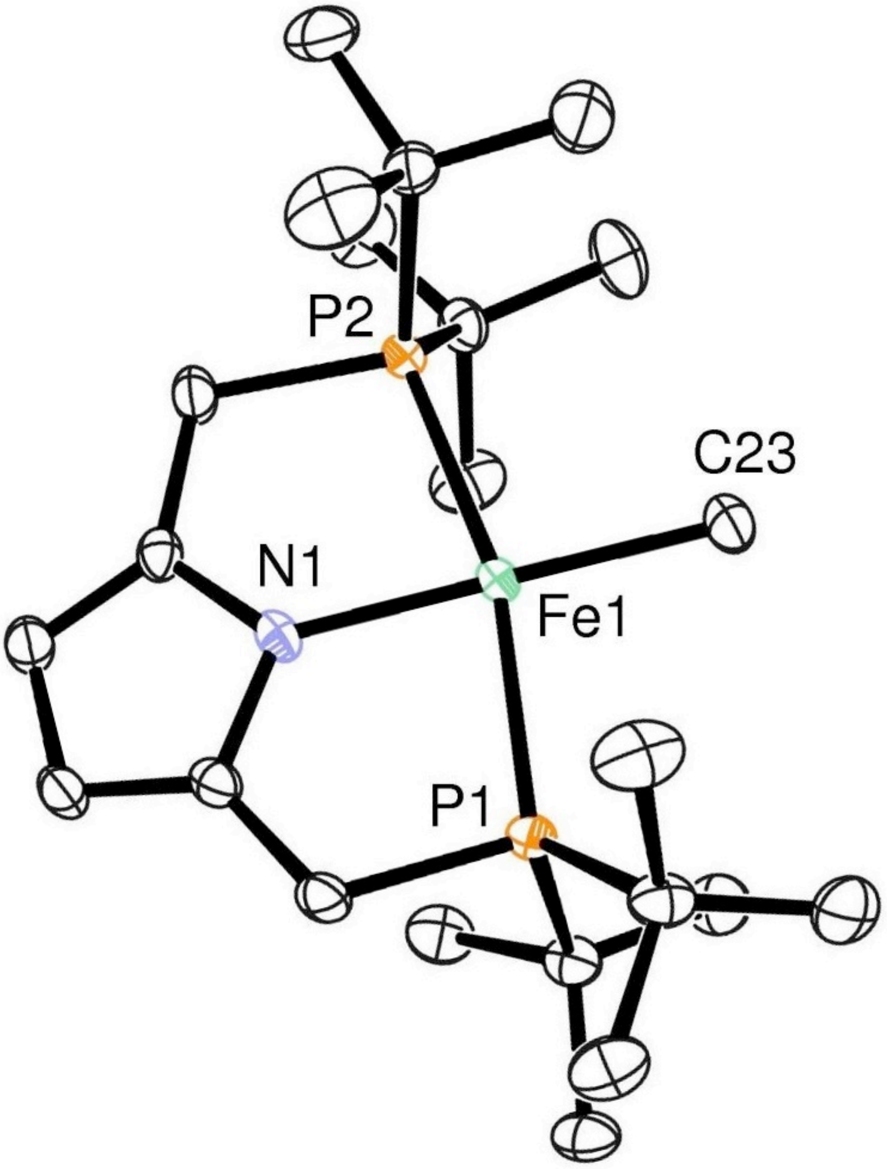

Supplementary Figure 6 | ORTEP drawing of 4. Thermal ellipsoids are shown at the with the 50% probability level. Hydrogen atoms are omitted for clarity. Selected bond lengths (Å) and angles (deg): Fe(1)–N(1) = 1.9266(18), Fe(1)–C(23) = 2.022(3), Fe(1)–P(1) = 2.2722(6), Fe(1)–P(2) = 2.2797(5), N(1)–Fe(1)–C(23) = 178.69(9), P1(1)–Fe(1)–P(2) = 164.71(2), N(1)–Fe(1)–P(1) = 82.61(5), C(23)–Fe(1)–P(1) = 96.41(8), N(1)–Fe(1)–P(2) = 83.16(5), C(23)–Fe(1)–P(2) = 97.89(8).

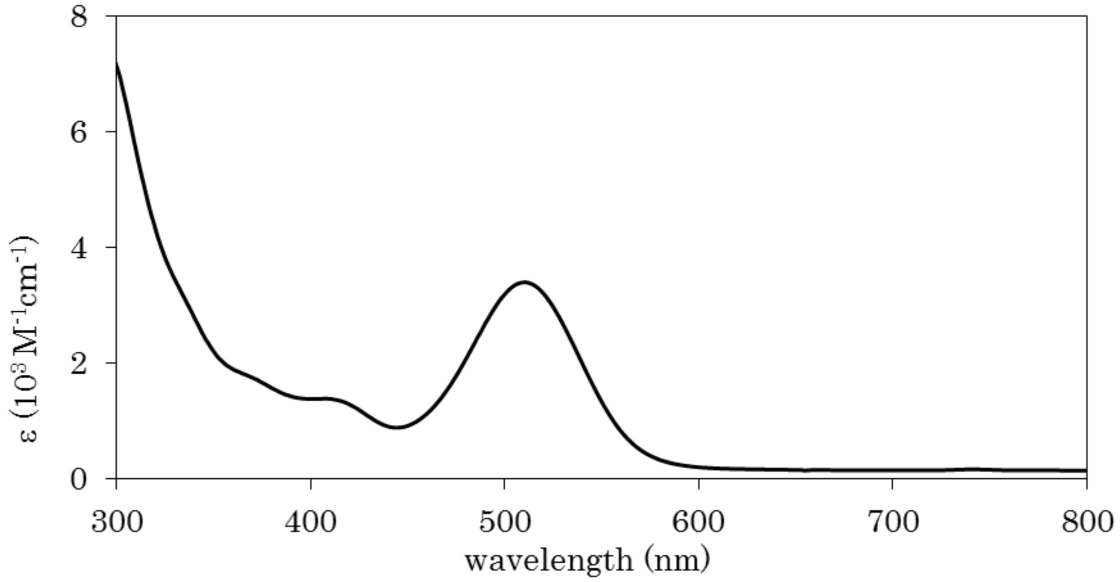

Supplementary Figure 7 | UV-Vis spectra of the dinitrogen complex 1 in THF (0.2 mM). The absorption spectra of **1** shows the absorption edge at *ca.* 600 nm. According to the absorption edge, the energy of HOMO-LUMO gap of **1** was calculated to be *ca.* 2.1 eV. This result is in good agreement with the result of the CVs of **1**.

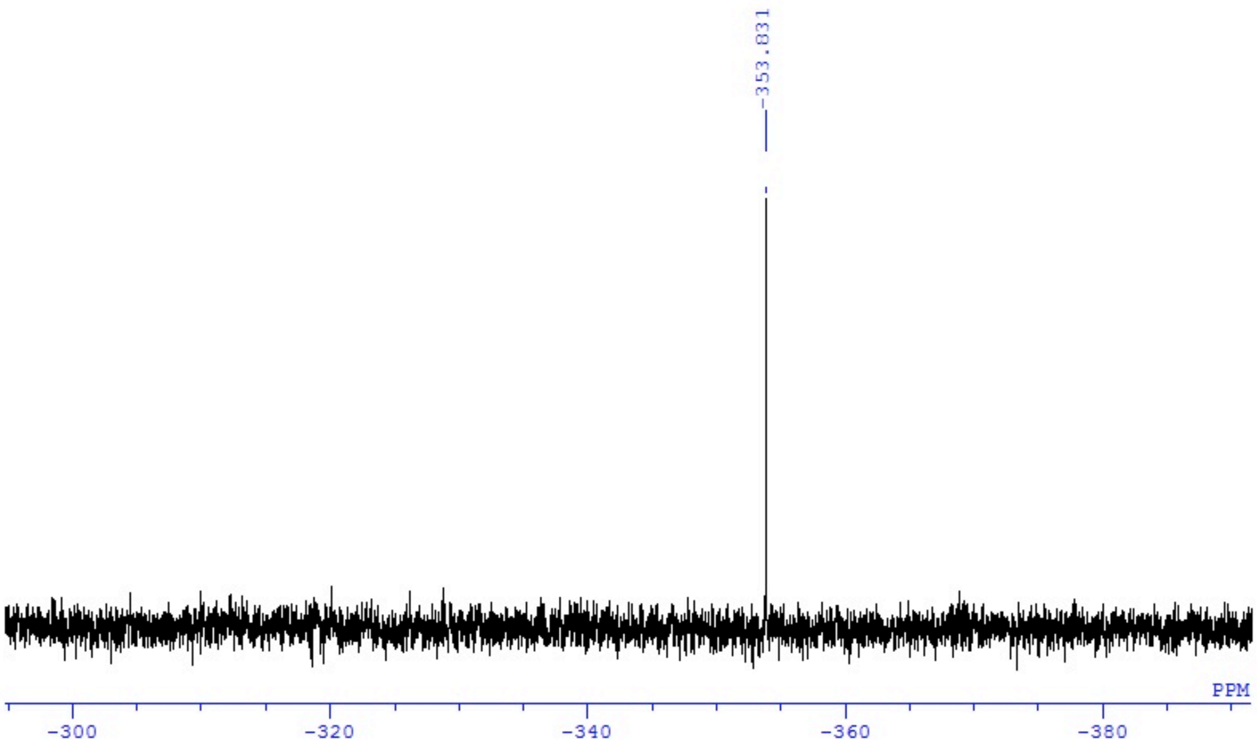

Supplementary Figure 8 | The $^{15}N\{^{1}H\}$ NMR spectrum of $^{15}NH_4Cl$ obtained from the reaction of 4, KC_8, and $[H(OEt_2)_2]BAr^F_4$ in Et_2O under $^{15}N_2$.

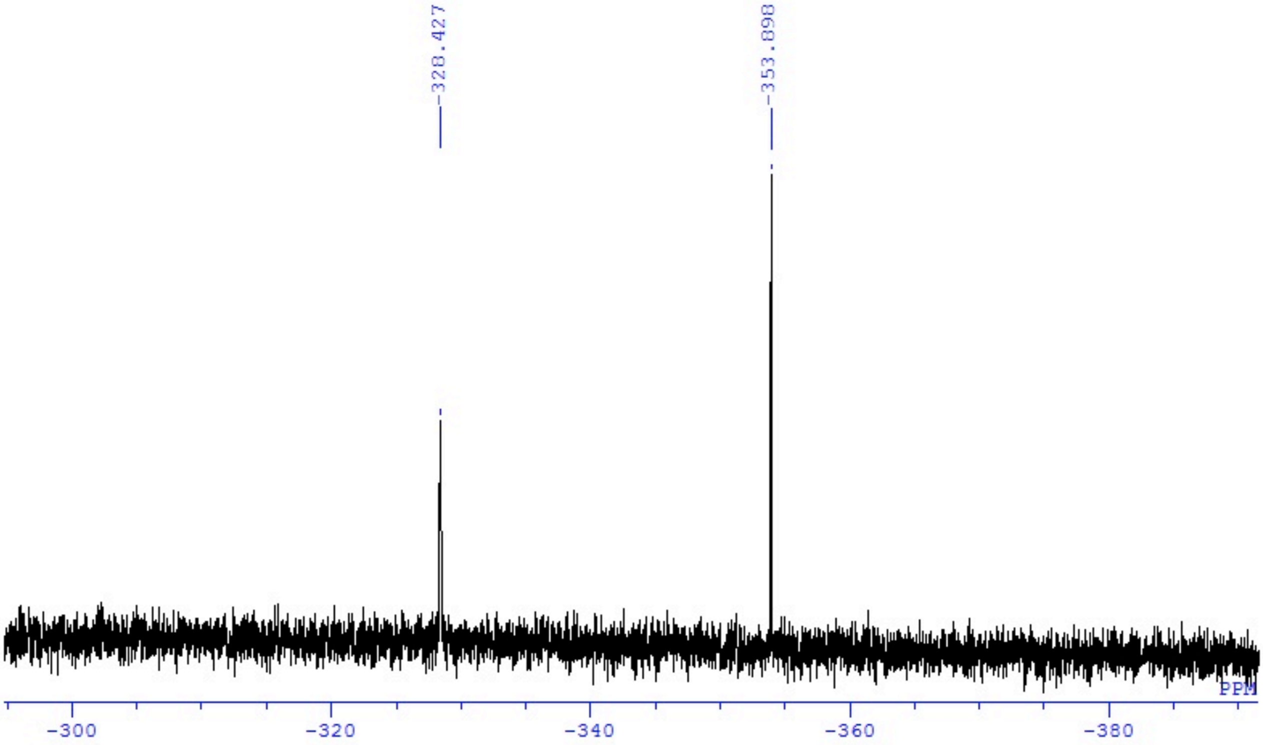

Supplementary Figure 9 | The $^{15}N\{^1H\}$ NMR spectrum of a mixture of $^{15}NH_4Cl$ and $^{15}NH_2{}^{15}NH_3Cl$ obtained from the reaction of 1, KC_8, and $[H(OEt_2)_2]BAr^F{}_4$ in THF under $^{15}N_2$.

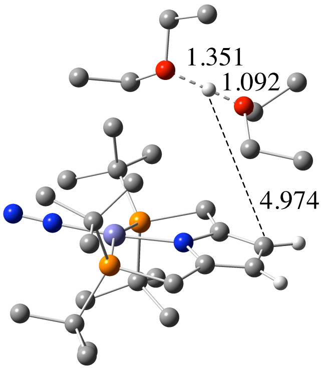

1-$H^+(OEt_2)_2$
doublet

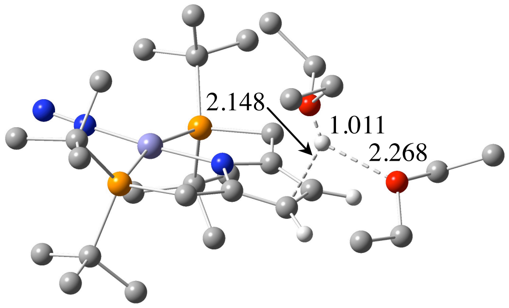

$\mathbf{TS_{II}}$
doublet

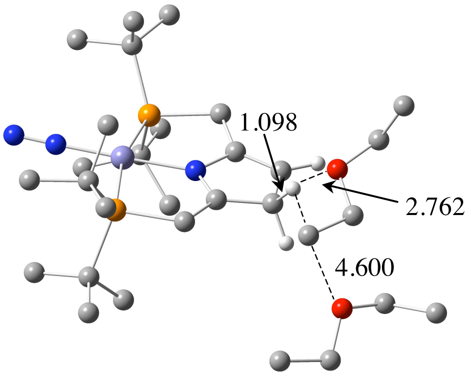

II-$2Et_2O$
doublet

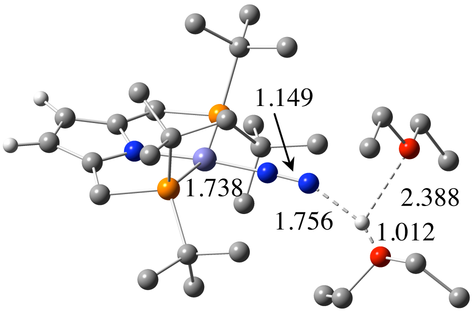

$\mathbf{TS_{III}}$
doublet

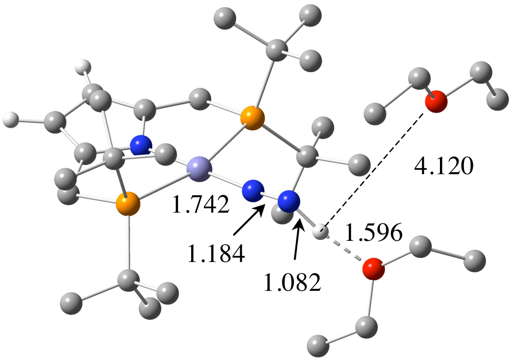

III-$2Et_2O$
doublet

Supplementary Figure 10 | Optimized structures of intermediates and transition states for protonation of 1. Selected interatomic distances are presented in Å. Hydrogen atoms are omitted except a transferring proton and hydrogen atoms at the ß-C atom to be attacked in the pyrrole ring.

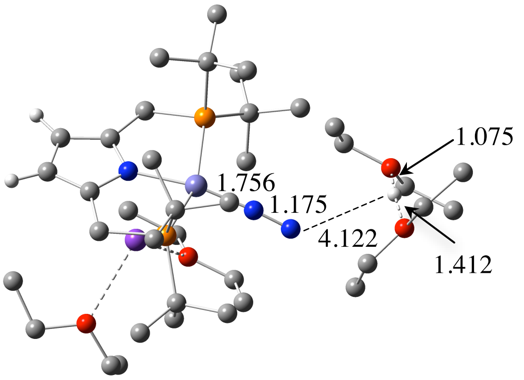

V-H$^+$(OEt$_2$)$_2$
singlet

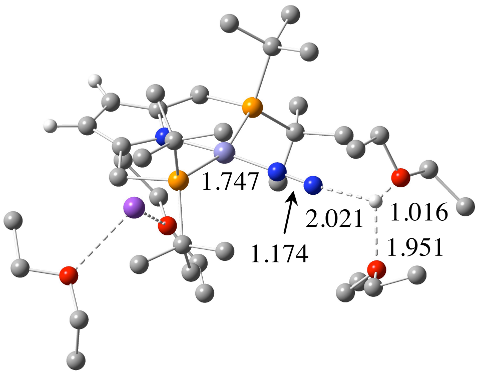

TS$_{VI}$
singlet

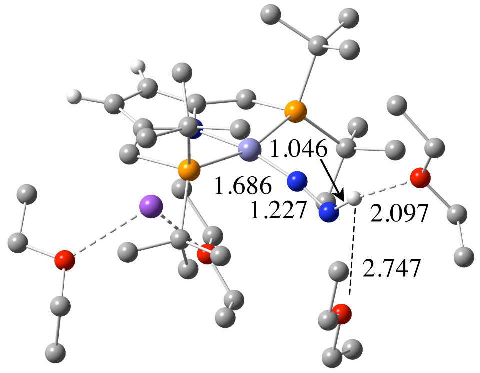

VI-2Et$_2$O
singlet

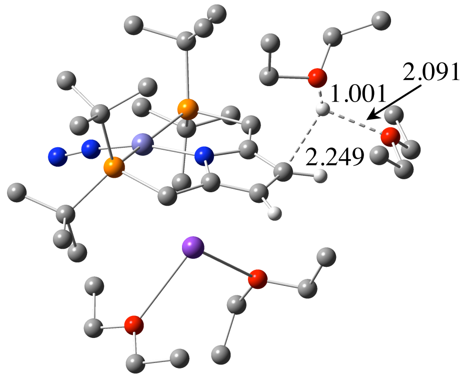

TS$_{VII}$
singlet

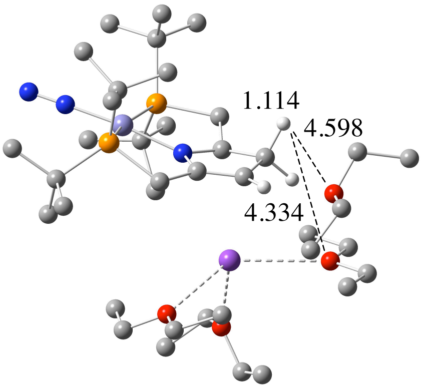

VII-2Et$_2$O
singlet

Supplementary Figure 11 | Optimized structures of intermediates and transition states for protonation of V. Selected interatomic distances are presented in Å. Hydrogen atoms are omitted except a transferring proton and hydrogen atoms at the ß-C atom to be attacked in the pyrrole ring.

Supplementary Table 1 | X-ray crystallographic data for 1 and 2.

	**1**	**2**
chemical formula	$C_{22}H_{42}FeN_3P_2$	$C_{22}H_{42}ClFeNP_2$
CCDC number	1447087	1447088
formula weight	466.39	473.83
dimensions of crystals	0.30 × 0.20 × 0.15	0.25 × 0.20 × 0.15
crystal system	monoclinic	orthorhombic
space group	$P2_1/a$	$P2_12_12_1$
a, Å	14.5154(9)	11.4325(5)
b, Å	12.9571(8)	14.4826(7)
c, Å	15.1849(9)	15.0623(7)
α, deg	90	90
β, deg	118.2775(12)	90
γ, deg	90	90
V, Å^3	2515.1(3)	2493.9(2)
Z	4	4
ρ_{calcd}, g cm^{-3}	1.232	1.262
$F(000)$	1004	1016.00
μ, cm^{-1}	7.386	8.472
trans. factors range	0.688-0.895	0.669-0.881
no. reflections measured	24235	24652
no. unique reflections	5735 (R_{int} = 0.0360)	5694(R_{int} = 0.0572)
no. parameters refined	295	287
$R1$ ($I > 2\sigma(I)$)[a]	0.0315	0.0300
$wR2$ (all data)[b]	0.0772	0.0684
GOF (all data)[c]	0.993	1.005
Flack parameters		0.024(12)
max diff peak / hole, e Å^{-3}	0.49/-0.35	0.66/-0.56

[a] $R1 = \Sigma||F_o|-|F_c||/\Sigma|F_o|$. [b] $wR2 = [\Sigma w(F_o^2-F_c^2)^2/\Sigma w(F_o^2)^2]^{1/2}$, $w = 4F_o^2/q\sigma(F_o^2)$ [q = 6.5 (**1**); q = 2 (**2**)]. [c] GOF = $[\Sigma w(F_o^2-F_c^2)^2/(N_o-N_{params})]^{1/2}$.

Supplementary Table 2 | X-ray crystallographic data for 3 and 4.

	**3**	**4**
chemical formula	$C_{22}H_{43}FeNP_2$	$C_{23}H_{45}FeNP_2$
CCDC number	1447089	1447090
formula weight	439.38	453.41
dimensions of crystals	0.20 × 0.20 × 0.20	0.30 × 0.30 × 0.20
crystal system	orthorhombic	orthorhombic
space group	$P2_12_12_1$	$P2_12_12_1$
a, Å	14.864(5)	11.4793(4)
b, Å	14.829(4)	14.5530(5)
c, Å	11.258(3)	15.0503(6)
α, deg	90	90
β, deg	90	90
γ, deg	90	90
V, Å^3	2481.3(13)	2514.28(15)
Z	4	4
ρ_{calcd}, g cm^{-3}	1.176	1.198
F(000)	952	984
μ, cm^{-1}	7.425	7.347
trans. factors range	0.489-0.862	0.644-0.863
no. reflections measured	22453	23735
no. unique reflections	5565 (R_{int} = 0.0708)	5752 (R_{int} = 0.0835)
no. parameters refined	282	290
$R1$ ($I > 2\sigma(I)$)[a]	0.0361	0.0352
$wR2$ (all data)[b]	0.0839	0.0882
GOF (all data)[c]	1.016	1.013
Flack parameters	-0.000(14)	0.007(13)
max diff peak / hole, e Å^{-3}	0.83/-0.70	0.61/-0.45

[a] $R1 = \Sigma||F_o|-|F_c||/\Sigma|F_o|$. [b] $wR2 = [\Sigma w(F_o^2-F_c^2)^2/\Sigma w(F_o^2)^2]^{1/2}$, $w = 4F_o^2/q\sigma(F_o^2)$ [q = 1.6 (**3**); q = 1.5 (**4**)]. [c] GOF = $[\Sigma w(F_o^2-F_c^2)^2/(N_o-N_{params})]^{1/2}$.

Supplementary Table 3 | Catalyst screening in reduction of dinitrogen into ammonia and hydrazine.

$$\underset{(1\ \text{atm})}{N_2} + \underset{(40\ \text{equiv})}{KC_8} + \underset{(38\ \text{equiv})}{[H(OEt_2)_2]BAr^F{}_4} \xrightarrow[\substack{Et_2O \\ -78\ °C,\ 1\ h}]{\substack{\text{cat.} \\ (0.010\ \text{mmol})}} NH_3 + NH_2NH_2 + H_2$$

run	cat.	NH_3 (equiv)[a]	NH_3 (%)[b]	NH_2NH_2 (equiv)[a]	NH_2NH_2 (%)[b]	fixed N atom (equiv)[c]	H_2 (equiv)[a]	H_2 (%)[b]
1	**1**	4.4±0.2	35	0.2±0.2	2	4.8	1.2±0.1	6
2	**2**	1.1±0.6	9	0	0	1.1	2.3±0.7	12
3	**3**	3.0±1.0	24	0.1±0.1	1	3.2	2.0±0.5	11
4	**4**	3.7±0.5	29	<0.1	<1	3.7	2.0±0.7	11
4	**5**	2.6±0.2	21	<0.1	<1	3.7	2.7±0.2	11
5	PNP-H	<0.1	<1	0	0	<0.1	3.8±0.5	20
6	none	0	0	0	0	0	3.9	21

[a] Equiv based on cat. [b] Yield based on $[H(OEt_2)_2]BAr^F{}_4$.
[c] Fixed N atom (equiv) = $[NH_3$ (equiv)] + $2[NH_2NH_2$ (equiv)].

Supplementary Table 4 | Screening of reductants, solvents, and temperature in reduction of dinitrogen into ammonia and hydrazine.

$$\underset{(1\ \text{atm})}{N_2} + \underset{(40\ \text{equiv})}{\text{reductant}} + \underset{(38\ \text{equiv})}{[H(OEt_2)_2]BAr^F{}_4} \xrightarrow[\text{solvent},\ -78\ °\text{C},\ 1\ \text{h}]{\text{cat. } \mathbf{1}\ (0.010\ \text{mmol})} NH_3 + NH_2NH_2 + H_2$$

run	solvent	reductant	NH_3 (equiv)[a]	NH_3 (%)[b]	NH_2NH_2 (equiv)[a]	NH_2NH_2 (%)[b]	fixed N atom (equiv)[c]	H_2 (equiv)[a]	H_2 (%)[b]
1	Et_2O	KC_8	4.4±0.2	35	0.2±0.2	2	4.8	1.2±0.1	6
2	Et_2O	none	0	0	0	0	0	0	0
3	Et_2O	$CoCp^*_2$	1.7	13	0.3	3	2.3	5.7	30
4	THF	KC_8	1.9±0.4	15	1.4±0.7	15	4.7	3.0±0.9	16
5	MeO^{t}Bu	KC_8	3.7	29	0.2	3	4.1	2.7	14
6	toluene	KC_8	0	0	0	0	0	2.1	11
7[d]	Et_2O	KC_8	0	0	0	0	0	5.2	27

[a] Equiv based on cat. [b] Yield based on $[H(OEt_2)_2]BAr^F{}_4$. [c] Fixed N atom (equiv) = [NH_3 (equiv)] + 2[NH_2NH_2 (equiv)]. [d] The reaction was carried out at rt for 1 h.

Supplementary Table 5 | Screening of the amounts of KC_8, $[H(OEt_2)_2]BAr^F{}_4$ and the catalyst 1 in reduction of dinitrogen into ammonia and hydrazine..

$$\underset{(1\ \text{atml})}{N_2} + \underset{(0.40\ \text{mmol})}{KC_8} + \underset{(0.38\ \text{mmol})}{[H(OEt_2)_2]BAr^F{}_4} \xrightarrow[\text{solvent, -78 °C, 1 h}]{\text{cat. } \mathbf{1}\ (x\ \text{mmol})} NH_3 + NH_2NH_2 + H_2$$

run	solvent	x (mmol)	NH_3 (mmol)	NH_3 (equiv)[a]	NH_2NH_2 (mmol)	NH_2NH_2 (equiv)[a]	fixed N atom (equiv)[b]	H_2 (mmol)	H_2 (equiv)[a]
1	Et_2O	0.010	0.044±0.002	4.4±0.2	0.002±0.002	0.2±0.2	4.8	0.012±0.001	1.2±0.1
2	Et_2O	0.0050	0.034	6.7	0.004	0.8	8.3	0.020	4.0
3	Et_2O	0.0025	0.027±0.001	10.9±0.4	0.004±0.001	1.6±0.2	14.1	0.020±0.001	8.0±0.8
4[c]	Et_2O	0.0025	0.036±0.001	14.3±0.3	0.005±0.001	1.8±0.2	17.9	0.031±0.04	12.3±1.7
5[d]	Et_2O	0.0025	0.032	12.7	0.002	0.8	14.3	0.096	38.4
6	THF	0.010	0.019±0.004	1.9±0.4	0.014±0.007	1.4±0.7	4.7	0.030±0.009	3.0±0.9
7	THF	0.0050	0.014±0.001	2.9±0.2	0.012±0.001	2.4±0.1	7.7	0.023±0.002	4.6±0.3
8	THF	0.0025	0.004	1.6	0.002	0.8	3.2	0.034	13.6

[a] Equiv based on cat. [b] Fixed N atom (equiv) = [NH_3 (equiv)] + 2[NH_2NH_2 (equiv)]. [c] 0.50 mmol of KC_8 and 0.46 mmol of $[H(OEt_2)_2]BAr^F{}_4$ were used. [d] 1.00 mmol of KC_8 and 0.92 mmol of $[H(OEt_2)_2]BAr^F{}_4$ were used.

Supplementary Table 6 | Reaction of hydrazine with iron complexes, KC_8, and $[H(OEt_2)_2]BAr^F_4$ under argon atmosphere.

$$NH_2NH_2 \;(4\ \text{equiv},\ 0.040\ \text{mmol}) + KC_8 + [H(OEt_2)_2]BAr^F_4 \xrightarrow[\text{Et}_2\text{O},\ -78\ °\text{C},\ 1\ \text{h},\ \text{Ar (1 atm)}]{\text{cat. (0.010 mmol)}} NH_3 + H_2$$

run	cat.	KC_8 (equiv)[a]	$[H(OEt_2)_2]BAr^F_4$ (equiv)[a]	NH_3 (equiv)[a]	H_2 (equiv)[a]	NH_2NH_2 (equiv)[a]	NH_2NH_2 (%)[b]
1	**4**	40	38	1.2	3.3	2.5	63
2	**4**	0	38	<0.1	0	3.3	83
3	**4**	0	1	0	0	2.8	71
4	**4**	0	0	<0.1	0	3.4	85
5	**3**	0	0	<0.1	0.1	3.4	85
6	**1**	0	0	<0.1	0	2.5	62
7	**1**	0	1	<0.1	0	2.3	57
8	none	40	38	<0.1	2.9	3.0	75
9[c]	**4**	40	38	0.4	4.2	3.2	80

[a] Equiv based on cat. [b] Recovery of NH_2NH_2. [c] THF was used as a solvent instead of Et_2O.

Supplementary Table 7 | Catalytic reduction of dinitrogen into N(SiMe$_3$).[a]

$$\underset{(1\ \text{atm})}{N_2} + \underset{(600\ \text{equiv})}{6\ \text{Na}} + \underset{(600\ \text{equiv})}{6\ \text{Me}_3\text{SiCl}} \xrightarrow[\text{THF, rt, 20 h}]{\text{cat. (0.020 mmol)}} 2\ \text{N(SiMe}_3)_3 \xrightarrow[\text{rt}]{H_2SO_4} 2\ NH_3$$

run	cat.	$N(SiMe_3)_3$ (mmol)[b]	$N(SiMe_3)_3$ (equiv)[b]
1	**1**	0.67±0.04 (0.42±0.14)	33 (21)
2	**2**	0.28±0.01 (0.25±0.01)	14 (13)
3	**3**	0.07±0.01 (0.09±0.01)	4 (4)
4	**4**	0.06±0.01 (0.08±0.01)	3 (4)

[a] Averages of two runs are shown. [b] Amount of $N(SiMe_3)_3$ was determined by GC. Amount of NH_3 after hydrolysis is in parenthesis.

Supplementary Table 8 | Effect of solvent and reductant on catalytic reduction of dinitrogen into $N(SiMe_3)$.[a]

$$\underset{(1\ \mathrm{atm})}{N_2} + \underset{(600\ \mathrm{equiv})}{6\ \mathrm{reductant}} + \underset{(600\ \mathrm{equiv})}{6\ Me_3SiCl} \xrightarrow[\text{solvent, rt, 20 h}]{\text{cat.}\mathbf{1}\ (0.020\ \mathrm{mmol})} 2\ N(SiMe_3)_3 \xrightarrow[\text{rt}]{H_2SO_4} 2\ NH_3$$

run	solvent	reductant	$N(SiMe_3)_3$ (equiv)[a]
1	THF	Na	33 (21)
2	DME	Na	0 (n.d.)[b]
3[c]	THF	KC_8	10 (12)

[a]Amount of NH_3 after hydrolysis is in parenthesis. [b] n.d. = not determined. [c] 0.010 mmol of **1** was used.

Supplementary Table 9 | Selected geometric parameters of 1. Bond distances and angles are presented in Å and degrees

	Doublet				Quartet			
	Fe–N	N–N	N(py)–Fe–N	P–Fe–P	Fe–N	N–N	N(py)–Fe–N	P–Fe–P
B3LYP	1.783	1.134	176.8	163.7	1.976	1.128	148.3	154.3
B3LYP-D3	1.779	1.135	176.3	162.9	1.987	1.127	147.7	154.4
M06	1.773	1.138	176.8	164.2	1.881	1.134	146.3	146.1
Expt.	1.764	1.134	176.8	164.2				

Supplementary Table 10 | Relative energies of the doublet and quartet spin states of 1.

Energies are presented in kcal/mol

	Doublet	Quartet
B3LYP	0.0	+7.1
B3LYP-D3	0.0	+9.2
M06	+1.2	0.0

Supplementary Table 11 | Cartesian coordinates of optimized intermediates and transition states of 1 in the doublet state.

SCF energy = -1834.68512469 hartree

ZPE = 0.616490 hartree

Atom	Coordinates (Angstroms)		
	X	Y	Z
Fe	-0.000003	-0.343123	-0.127170
P	2.258324	-0.004996	-0.103230
P	-2.258336	-0.005015	-0.103210
N	-0.000014	1.425817	-0.929580
N	0.000003	-2.006714	0.502673
N	0.000091	-3.078610	0.874930
C	1.112245	2.220269	-1.146430
C	0.713269	3.507217	-1.464885
C	-0.713318	3.507209	-1.464889
C	-1.112280	2.220261	-1.146416
C	2.461466	1.567752	-1.094404
C	3.376053	-1.272823	-0.976749
C	2.568708	-1.703350	-2.223190
C	4.739618	-0.715276	-1.429711
C	3.595159	-2.509186	-0.083875
C	2.890105	0.440308	1.636480
C	2.158460	1.741836	2.035357
C	2.454873	-0.663288	2.623298
C	4.408334	0.661675	1.725558
C	-2.461494	1.567732	-1.094383
C	-3.376055	-1.272846	-0.976751

C	-2.568743	-1.703263	-2.223250
C	-4.739657	-0.715331	-1.429638
C	-3.595080	-2.509281	-0.083957
C	-2.890100	0.440295	1.636508
C	-4.408327	0.661664	1.725616
C	-2.454837	-0.663273	2.623346
C	-2.158460	1.741834	2.035356
H	1.362723	4.343704	-1.694540
H	-1.362780	4.343689	-1.694547
H	3.240146	2.217622	-0.678900
H	2.801394	1.281090	-2.098698
H	2.352241	-0.859245	-2.887928
H	3.144227	-2.441311	-2.797537
H	1.613592	-2.158253	-1.939243
H	5.286651	-1.498136	-1.972179
H	4.632896	0.136545	-2.108755
H	5.363169	-0.401745	-0.589407
H	2.652482	-2.920032	0.290166
H	4.091553	-3.294373	-0.669455
H	4.239527	-2.286204	0.772170
H	2.445581	2.598422	1.419420
H	1.072885	1.631259	1.950616
H	2.396259	1.980752	3.080179
H	2.923397	-1.628440	2.415534
H	2.734599	-0.370783	3.644014
H	1.369639	-0.807326	2.596914
H	4.667369	1.027925	2.727999
H	4.966868	-0.264581	1.561750
H	4.757681	1.406755	1.002293

H	-2.801421	1.281064	-2.098676
H	-3.240180	2.217596	-0.678878
H	-1.613601	-2.158153	-1.939370
H	-3.144259	-2.441204	-2.797625
H	-2.352331	-0.859105	-2.887939
H	-5.286660	-1.498174	-1.972160
H	-5.363210	-0.401901	-0.589298
H	-4.632992	0.136552	-2.108614
H	-2.652378	-2.920079	0.290074
H	-4.239483	-2.286408	0.772087
H	-4.091401	-3.294469	-0.669598
H	-4.667341	1.027919	2.728060
H	-4.757688	1.406742	1.002355
H	-4.966867	-0.264591	1.561824
H	-2.923367	-1.628431	2.415631
H	-1.369604	-0.807312	2.596931
H	-2.734528	-0.370733	3.644062
H	-2.396229	1.980751	3.080186
H	-1.072886	1.631268	1.950582
H	-2.445611	2.598414	1.419425

Supplementary Table 12 | Cartesian coordinates of optimized intermediates and transition states of 1 in the quartet state.

SCF energy = -1834.66728168 hartree

ZPE = 0.613229 hartree

Atom	Coordinates (Angstroms)		
	X	Y	Z
Fe	0.003276	-0.551852	-0.154747
P	2.325778	-0.023310	-0.034415
P	-2.329773	-0.047880	-0.053778
N	-0.008410	1.046442	-1.402158
N	0.026810	-2.529373	0.042372
N	0.036119	-3.536266	0.549568
C	1.087891	1.856631	-1.620472
C	0.670623	3.111978	-2.034091
C	-0.755994	3.074435	-2.076498
C	-1.130761	1.798784	-1.686479
C	2.456165	1.287645	-1.377436
C	3.621292	-1.319969	-0.564190
C	2.995206	-2.050366	-1.775113
C	4.981300	-0.738190	-0.998500
C	3.832437	-2.343860	0.567256
C	2.835629	0.872394	1.570521
C	1.978350	2.154549	1.629492
C	2.433996	-0.017174	2.765909
C	4.320705	1.252530	1.675728
C	-2.478898	1.171678	-1.475586
C	-3.623038	-1.387647	-0.467343

C	-3.024717	-2.194541	-1.643364
C	-4.996999	-0.844304	-0.906549
C	-3.788489	-2.333808	0.737018
C	-2.818778	0.951145	1.496672
C	-4.319102	1.234360	1.670545
C	-2.278551	0.188610	2.724844
C	-2.073472	2.301651	1.404538
H	1.306988	3.954330	-2.280422
H	-1.420217	3.885112	-2.353655
H	3.186308	2.061105	-1.112606
H	2.847921	0.786969	-2.272946
H	2.799770	-1.372169	-2.612756
H	3.688796	-2.823902	-2.130556
H	2.051868	-2.536375	-1.512473
H	5.622491	-1.553278	-1.361250
H	4.879177	-0.017758	-1.816135
H	5.510395	-0.246792	-0.180090
H	2.882000	-2.749765	0.930940
H	4.433020	-3.184129	0.194435
H	4.370686	-1.910501	1.416016
H	2.213981	2.862991	0.830892
H	0.912461	1.922163	1.551691
H	2.144057	2.656683	2.591943
H	2.995490	-0.954559	2.802591
H	2.621055	0.520088	3.705306
H	1.366382	-0.266921	2.728445
H	4.479048	1.873680	2.567726
H	4.961979	0.371727	1.774751
H	4.658989	1.829802	0.808363

H	-2.803447	0.594502	-2.351606
H	-3.258054	1.918350	-1.285099
H	-2.066976	-2.649431	-1.378909
H	-3.718309	-2.999803	-1.919567
H	-2.868173	-1.576063	-2.533852
H	-5.635902	-1.683421	-1.214273
H	-5.517990	-0.314885	-0.107124
H	-4.913351	-0.168215	-1.763726
H	-2.822990	-2.718533	1.084125
H	-4.287629	-1.845734	1.579812
H	-4.404974	-3.195070	0.446912
H	-4.466387	1.903343	2.529331
H	-4.743023	1.731633	0.791192
H	-4.894936	0.325045	1.865090
H	-2.739184	-0.796306	2.846023
H	-1.193946	0.041639	2.647130
H	-2.476484	0.765635	3.638002
H	-2.150571	2.820813	2.368999
H	-1.014060	2.173174	1.168272
H	-2.491890	2.956027	0.635484

Supplementary Table 13 | Cartesian coordinates of optimized intermediates and transition states of 1-H^{+}(OEt$_2$)$_2$.

SCF energy = -2302.50001866 hartree

ZPE = 0.908089 hartree

Atom	Coordinates (Angstroms)		
	X	Y	Z
C	-0.388584	1.051906	3.317117
C	-0.321683	-0.359405	3.524839
C	0.150456	-0.917645	2.343743
N	0.374035	0.085070	1.416347
C	0.047655	1.287326	2.019094
C	0.529102	-2.330294	1.994315
P	1.073361	-2.362951	0.197160
C	2.763362	-3.236107	0.246289
C	2.820487	-4.505558	1.117683
Fe	1.205512	-0.129455	-0.335563
N	1.817401	-0.289819	-1.990318
N	2.176815	-0.378173	-3.063982
C	0.269429	2.555839	1.247951
P	1.463646	2.143682	-0.132249
C	3.186621	2.516097	0.591658
C	3.271926	1.762478	1.938979
C	-0.250124	-3.387686	-0.705322
C	-1.606657	-2.752600	-0.333102
C	-0.270622	-4.879274	-0.334444
C	-0.049910	-3.225332	-2.225587
C	0.992426	3.339262	-1.532466

C	-0.283079	2.735861	-2.159543
C	0.680939	4.771911	-1.056822
C	2.095557	3.378166	-2.607319
C	4.255277	1.922504	-0.350462
C	3.464478	4.008918	0.831187
C	3.224295	-3.576807	-1.183768
C	3.726452	-2.181740	0.838894
O	-3.900065	0.558573	0.935274
C	-3.310458	1.908929	1.107259
C	-4.358555	2.850713	1.656584
C	-3.377704	-0.531667	1.797902
C	-3.850688	-0.343726	3.220335
O	-3.897423	-0.196367	-1.387745
C	-5.204674	-0.069688	-2.015096
C	-6.232644	-0.705469	-1.097646
C	-2.775393	0.362746	-2.129068
C	-2.424669	-0.458991	-3.355893
H	-0.705060	1.801163	4.033877
H	-0.580476	-0.895670	4.430443
H	0.645444	3.368294	1.879152
H	-0.648296	2.928323	0.774158
H	-1.107840	2.685157	-1.439471
H	-0.613544	3.366180	-2.995335
H	-0.101742	1.726280	-2.542538
H	0.368100	5.373405	-1.920504
H	-0.135282	4.795199	-0.327187
H	1.549562	5.260423	-0.611255
H	2.382675	2.375300	-2.938291
H	1.723429	3.927209	-3.481983

H	2.990721	3.896387	-2.252179
H	2.579909	2.161396	2.686154
H	3.056143	0.695658	1.821636
H	4.290858	1.864051	2.333816
H	4.287659	2.421899	-1.321596
H	5.244390	2.030171	0.112903
H	4.077531	0.856103	-0.524723
H	4.426950	4.116704	1.348187
H	3.529920	4.568103	-0.106244
H	2.697191	4.473718	1.460268
H	-0.280325	-3.046971	2.173518
H	1.368440	-2.666466	2.611834
H	-1.605418	-1.672034	-0.506220
H	-2.395683	-3.190718	-0.957031
H	-1.874353	-2.927143	0.713016
H	-1.147684	-5.354930	-0.793333
H	0.616706	-5.399855	-0.705259
H	-0.336189	-5.032646	0.748459
H	-0.056552	-2.171823	-2.517940
H	0.887294	-3.665731	-2.572442
H	-0.870449	-3.729018	-2.753328
H	3.848269	-4.891406	1.110626
H	2.557234	-4.306745	2.161323
H	2.168479	-5.297444	0.744610
H	2.646863	-4.402888	-1.609552
H	3.149071	-2.716940	-1.856883
H	4.275625	-3.890905	-1.156120
H	4.736300	-2.606456	0.903602
H	3.775786	-1.287327	0.207843

H	3.431351	-1.868888	1.846788
H	-2.448385	1.789976	1.765492
H	-2.962216	2.203835	0.114692
H	-3.787355	-1.431848	1.337269
H	-2.289475	-0.534018	1.715481
H	-1.953277	0.360978	-1.408623
H	-3.008341	1.403298	-2.385566
H	-5.406808	0.994983	-2.187662
H	-5.165567	-0.581388	-2.979703
H	-3.902108	0.238848	-0.108967
H	-1.478652	-0.091501	-3.767740
H	-2.298240	-1.510813	-3.086859
H	-3.186493	-0.377429	-4.136406
H	-7.220089	-0.650586	-1.566202
H	-5.989966	-1.758412	-0.922703
H	-6.280899	-0.187967	-0.134019
H	-3.920985	3.850615	1.750909
H	-5.223258	2.913433	0.989218
H	-4.696535	2.526527	2.644777
H	-3.493397	-1.190403	3.814858
H	-3.432313	0.565717	3.660065
H	-4.942999	-0.309282	3.271559

Supplementary Table 14 | Cartesian coordinates of optimized intermediates and transition states of TS_{II}.

SCF energy = -2302.49166611 hartree

ZPE = 0.908445 hartree

Imaginary frequency: 160i cm^{-1}

Atom	X	Y	Z
C	-1.503820	-1.787294	-1.613420
C	-0.309384	-1.682101	-0.918281
N	-0.078747	-0.355906	-0.592979
C	-1.128908	0.386206	-1.083442
C	-2.047678	-0.461472	-1.705194
C	0.671063	-2.713661	-0.437116
P	2.264900	-1.809296	-0.054845
C	3.299121	-1.940599	-1.649923
C	3.932195	-3.320386	-1.893252
Fe	1.541880	0.349561	0.240037
P	0.510487	2.364406	-0.124445
C	1.346618	3.310087	-1.546746
C	0.669081	4.642867	-1.901239
C	-1.163585	1.862264	-0.808704
N	2.956565	0.957203	1.121657
N	3.843116	1.339090	1.717175
C	0.083872	3.475585	1.357586
C	1.347999	4.197493	1.859614
C	-1.023024	4.514313	1.093814
C	-0.394506	2.493053	2.450672

C	3.037054	-2.769875	1.389677
C	4.499901	-2.332831	1.599497
C	2.217755	-2.351628	2.631236
C	2.963263	-4.301951	1.241718
C	2.831550	3.547823	-1.201602
C	1.299732	2.358453	-2.762651
C	4.388759	-0.849111	-1.591552
C	2.358631	-1.618484	-2.833098
C	-6.176859	-0.667083	-0.979370
C	-5.904391	-0.044146	-2.342654
O	-4.992094	-1.265523	-0.433808
C	-4.852843	-2.668359	-0.688707
C	-5.608971	-3.506765	0.336039
O	-2.908918	-0.223952	1.204106
C	-4.124226	0.353354	1.850590
C	-4.415486	1.693335	1.218429
C	-2.357409	-1.474527	1.823052
C	-1.319279	-1.088808	2.848523
H	-2.941896	-0.164634	-2.237510
H	-1.935227	-2.694127	-2.018798
H	-1.914818	2.093809	-0.047768
H	-1.429519	2.452358	-1.692260
H	0.281530	2.157537	-3.109024
H	1.853233	2.812250	-3.594220
H	1.773060	1.399463	-2.527577
H	1.113241	5.041320	-2.822874
H	-0.406535	4.526656	-2.073853
H	0.814806	5.391596	-1.116867
H	3.345442	2.604665	-0.990943

H	3.326402	4.018547	-2.060877
H	2.964845	4.206217	-0.340436
H	-1.279814	1.927244	2.139947
H	0.390265	1.772797	2.707124
H	-0.655357	3.054208	3.357248
H	1.659336	4.987703	1.170477
H	1.131667	4.669661	2.826473
H	2.186498	3.509289	2.002478
H	-1.245422	5.041589	2.030856
H	-0.721116	5.262542	0.358687
H	-1.954133	4.055208	0.748142
H	0.816202	-3.529950	-1.152803
H	0.330431	-3.173819	0.500024
H	1.817254	-0.680352	-2.686026
H	2.960529	-1.524347	-3.745799
H	1.618932	-2.406411	-3.002425
H	4.419083	-3.321814	-2.877306
H	4.695533	-3.566092	-1.150269
H	3.179623	-4.116617	-1.895772
H	3.940463	0.145650	-1.485523
H	5.083458	-0.992620	-0.759481
H	4.970733	-0.864457	-2.521955
H	3.365296	-4.767014	2.151488
H	1.933187	-4.654230	1.122877
H	3.549965	-4.669615	0.397867
H	5.151665	-2.671017	0.789245
H	4.592739	-1.246563	1.687693
H	4.875164	-2.778009	2.530013
H	2.628205	-2.845581	3.521195

H	2.255492	-1.268989	2.791867
H	1.166816	-2.646251	2.543907
H	-6.980334	-1.411991	-1.040787
H	-6.496090	0.098109	-0.263055
H	-5.182000	-2.901047	-1.710946
H	-3.780373	-2.870690	-0.637156
H	-4.922770	-0.372198	1.704782
H	-3.850983	0.433930	2.902494
H	-3.209656	-2.017519	2.236873
H	-1.938612	-2.016361	0.976361
H	-3.018475	-0.350357	0.207247
H	-5.351391	2.074877	1.639589
H	-3.623129	2.414867	1.429496
H	-4.543976	1.599740	0.135610
H	-0.958957	-2.001320	3.334137
H	-0.472830	-0.590572	2.368885
H	-1.734225	-0.435619	3.622079
H	-6.820184	0.395177	-2.754413
H	-5.150044	0.746033	-2.258329
H	-5.535589	-0.796953	-3.047828
H	-5.454902	-4.574835	0.144620
H	-5.252747	-3.280580	1.347944
H	-6.685463	-3.307140	0.299227

Supplementary Table 15 | Cartesian coordinates of optimized intermediates and transition states of II-2Et_2O.

SCF energy = -2302.52701214 hartree

ZPE = 0.907075 hartree

Atom	Coordinates (Angstroms)		
	X	Y	Z
C	-0.577108	-2.505936	1.731787
C	0.563106	-1.922850	1.322481
N	0.300282	-0.579905	0.870056
C	-0.971686	-0.320875	1.027928
C	-1.693466	-1.511276	1.578298
C	1.994240	-2.361833	1.306013
P	2.961080	-1.262043	0.129170
C	3.039465	-2.239021	-1.493837
C	3.874826	-3.525883	-1.414002
Fe	1.613364	0.587757	-0.007664
P	-0.091386	2.111357	0.195170
C	-0.687187	3.095813	-1.308564
C	-2.138146	3.600596	-1.188499
C	-1.536979	0.987678	0.599111
N	2.779239	1.650313	-0.849462
N	3.509556	2.326113	-1.382258
C	0.146560	3.157986	1.760559
C	1.491203	3.906524	1.646389
C	-0.991635	4.151760	2.039571
C	0.258720	2.159171	2.935459
C	4.642299	-1.061003	0.981575

C	5.674305	-0.488615	-0.008304
C	4.390975	-0.020882	2.097543
C	5.197499	-2.356594	1.604605
C	0.254563	4.285719	-1.573783
C	-0.589621	2.100478	-2.488664
C	3.581640	-1.322088	-2.610567
C	1.572102	-2.578733	-1.838939
C	-3.777013	-0.326950	-3.106141
C	-4.683606	0.858071	-2.820748
O	-2.924464	-0.528302	-1.975361
C	-1.917971	-1.524083	-2.162073
C	-2.450331	-2.953801	-2.126456
O	-4.919806	-1.503118	1.922341
C	-5.266357	-0.670575	0.816159
C	-5.289483	0.769432	1.302218
C	-4.972439	-2.900081	1.637568
C	-6.388039	-3.469349	1.705400
H	-2.475244	-1.832809	0.877822
H	-0.689228	-3.506173	2.128767
H	-2.217013	1.402355	1.349577
H	-2.132634	0.821583	-0.306917
H	-1.256714	1.242063	-2.358803
H	-0.883615	2.611382	-3.414313
H	0.435598	1.733093	-2.614797
H	-2.423782	4.078660	-2.134461
H	-2.847934	2.788048	-1.007561
H	-2.256213	4.341939	-0.395192
H	1.304610	3.980355	-1.620245
H	-0.006555	4.735016	-2.540030

H	0.151993	5.062798	-0.810747
H	-0.680422	1.629658	3.126371
H	1.042605	1.413124	2.758727
H	0.516153	2.712066	3.846887
H	1.495387	4.639720	0.836889
H	1.682980	4.444088	2.583326
H	2.320055	3.210125	1.478839
H	-0.831182	4.616895	3.020556
H	-1.019927	4.952571	1.295953
H	-1.971099	3.661639	2.059590
H	2.096998	-3.425456	1.073939
H	2.423266	-2.210920	2.302812
H	0.958047	-1.671525	-1.878924
H	1.535369	-3.053292	-2.827192
H	1.115243	-3.268307	-1.122124
H	3.772032	-4.082511	-2.354259
H	4.937140	-3.306116	-1.273697
H	3.546513	-4.182636	-0.600870
H	2.985846	-0.407216	-2.698850
H	4.623425	-1.035863	-2.452377
H	3.525756	-1.856511	-3.567442
H	6.151614	-2.128603	2.096685
H	4.531290	-2.773181	2.366373
H	5.387977	-3.129003	0.855675
H	5.964502	-1.228760	-0.759100
H	5.305337	0.404807	-0.521096
H	6.578634	-0.207734	0.545642
H	5.326201	0.165332	2.639742
H	4.041265	0.930308	1.680023

H	3.649420	-0.366574	2.827230
H	-3.159717	-0.137846	-3.998486
H	-4.374027	-1.229040	-3.298668
H	-1.378819	-1.334979	-3.103407
H	-1.210212	-1.370835	-1.343980
H	-4.529191	-0.798971	0.008446
H	-6.250841	-0.946875	0.410558
H	-4.523165	-3.101690	0.651255
H	-4.338314	-3.379167	2.392141
H	-2.226175	-1.282111	2.510340
H	-5.488519	1.450978	0.468112
H	-6.065480	0.909108	2.061957
H	-4.325081	1.037035	1.746731
H	-6.367297	-4.553502	1.544730
H	-6.827460	-3.271808	2.688881
H	-7.038201	-3.028163	0.942677
H	-5.362298	1.022693	-3.664436
H	-5.285580	0.677507	-1.924161
H	-4.095315	1.768186	-2.667156
H	-1.613085	-3.659796	-2.171275
H	-3.005014	-3.133421	-1.198876
H	-3.114785	-3.164212	-2.970380

Supplementary Table 16 | Cartesian coordinates of optimized intermediates and transition states of TS_{III}.

SCF energy = -2302.48542828 hartree

ZPE = 0.907198 hartree

Imaginary frequency: 65i cm^{-1}

Atom	Coordinates (Angstroms)		
	X	Y	Z
C	-5.175927	0.498685	-0.786617
C	-5.109101	-0.930375	-0.785330
C	-3.769189	-1.269893	-0.720441
N	-3.001910	-0.115464	-0.666269
C	-3.873113	0.962197	-0.722503
C	-3.059310	-2.592277	-0.761728
P	-1.349067	-2.305842	-0.066010
C	-1.443247	-2.833028	1.757780
C	-1.528472	-4.352485	1.974225
Fe	-1.118332	-0.026903	-0.242837
N	0.604758	0.024186	-0.021813
N	1.748885	0.037252	0.086712
C	-3.291894	2.345798	-0.784131
P	-1.520662	2.229756	-0.194683
C	-1.550047	2.874011	1.591172
C	-2.658036	2.073305	2.310802
C	-0.198871	-3.410324	-1.097283
C	-0.033756	-2.661229	-2.439789
C	-0.762223	-4.818410	-1.370705
C	1.183079	-3.532934	-0.425024

C	-0.533366	3.338469	-1.377265
C	-0.459502	2.534927	-2.696201
C	-1.185084	4.705732	-1.658271
C	0.898158	3.549888	-0.845378
C	-0.211363	2.536491	2.280104
C	-1.838079	4.379369	1.709690
C	-0.219228	-2.252794	2.497887
C	-2.710749	-2.166549	2.337399
O	4.147743	-0.296166	-1.111929
C	3.523906	-0.922294	-2.317759
C	2.774882	0.143377	-3.082520
C	5.300164	-1.031670	-0.490144
C	6.562287	-0.695218	-1.248540
O	4.060207	0.662814	1.733656
C	3.862445	2.075290	1.700683
C	4.674015	2.661110	0.556781
C	3.321774	0.020729	2.772489
C	3.534798	-1.479793	2.665697
H	-6.071207	1.106664	-0.847064
H	-5.943855	-1.619342	-0.842579
H	-3.862887	3.075942	-0.200549
H	-3.276586	2.718740	-1.815613
H	-1.449581	2.373066	-3.136536
H	0.144255	3.088309	-3.426772
H	0.002019	1.553805	-2.539712
H	-0.583819	5.238278	-2.406859
H	-2.197186	4.606488	-2.062568
H	-1.233687	5.333323	-0.766175
H	1.388191	2.604769	-0.591271

H	1.498699	4.042927	-1.620665
H	0.912524	4.193202	0.039144
H	-3.655751	2.270116	1.909226
H	-2.475071	0.996033	2.240901
H	-2.662218	2.347491	3.373200
H	0.632691	3.073725	1.842170
H	-0.271307	2.819493	3.338943
H	0.004169	1.463620	2.227503
H	-1.970285	4.640522	2.767697
H	-1.011239	4.982364	1.322419
H	-2.752866	4.665626	1.179546
H	-2.934310	-2.944578	-1.793557
H	-3.592793	-3.382581	-0.222975
H	0.398510	-1.666015	-2.290254
H	0.631612	-3.233810	-3.099228
H	-0.989750	-2.536856	-2.960823
H	-0.063664	-5.361289	-2.021026
H	-0.883279	-5.401314	-0.454962
H	-1.727577	-4.783755	-1.884707
H	1.596829	-2.555997	-0.159105
H	1.145853	-4.144198	0.481170
H	1.877707	-4.021592	-1.121562
H	-1.693178	-4.555489	3.040479
H	-2.361334	-4.797225	1.418981
H	-0.606189	-4.861631	1.680782
H	0.726559	-2.670717	2.143703
H	-0.170665	-1.164019	2.382802
H	-0.303071	-2.479290	3.568599
H	-2.730968	-2.326996	3.422572

H	-2.720250	-1.088237	2.152556
H	-3.631370	-2.582999	1.919330
H	4.360310	-1.329872	-2.882401
H	2.887208	-1.730284	-1.953584
H	5.308129	-0.653501	0.528411
H	5.040381	-2.092947	-0.506879
H	2.253858	0.264152	2.671013
H	3.660609	0.395538	3.750909
H	2.792219	2.290775	1.565864
H	4.172584	2.514425	2.661765
H	3.419681	-0.092015	-0.439467
H	2.975956	-1.996878	3.452483
H	3.181413	-1.848922	1.696488
H	4.595737	-1.731561	2.771002
H	4.565009	3.750568	0.539621
H	5.736484	2.421532	0.675271
H	4.335684	2.267171	-0.407098
H	2.319363	-0.319927	-3.964079
H	1.978775	0.578785	-2.475825
H	3.452441	0.935432	-3.413132
H	7.398928	-1.198037	-0.751751
H	6.533378	-1.038665	-2.286733
H	6.745322	0.382791	-1.229588

Supplementary Table 17 | Cartesian coordinates of optimized intermediates and transition states of III-2Et$_2$O.

SCF energy = -2302.48924462 hartree

ZPE = 0.907148 hartree

Atom	Coordinates (Angstroms)		
	X	Y	Z
C	-3.272024	3.696755	-1.151018
C	-4.338504	2.813739	-0.782952
C	-3.778050	1.574857	-0.551743
N	-2.399616	1.650976	-0.742846
C	-2.108147	2.957790	-1.125453
C	-4.389308	0.241977	-0.238194
P	-3.017813	-0.881809	0.340482
C	-3.229649	-1.050332	2.213669
C	-4.491604	-1.839822	2.598312
Fe	-1.123202	0.335729	-0.214553
N	0.054536	-0.887806	0.171991
N	0.843281	-1.703339	0.510859
C	-0.695340	3.290029	-1.497506
P	0.400517	2.020683	-0.686463
C	0.982541	2.800019	0.938456
C	-0.292753	3.314451	1.643070
C	-3.222635	-2.493274	-0.631531
C	-2.701421	-2.159777	-2.048585
C	-4.680597	-2.983720	-0.727159
C	-2.350676	-3.604093	-0.017472
C	1.788304	1.659717	-1.918418

C	1.170631	0.700863	-2.961766
C	2.304173	2.930243	-2.624235
C	2.963859	0.947987	-1.226686
C	1.608054	1.709591	1.833087
C	1.968468	3.963874	0.740396
C	-1.979339	-1.710710	2.830915
C	-3.318024	0.392428	2.759581
O	2.585419	-2.657269	-1.271319
C	2.235388	-3.780234	-2.102195
C	0.802682	-3.602882	-2.572930
C	3.876442	-2.748490	-0.625182
C	5.024110	-2.500996	-1.594140
O	4.504517	-0.357808	1.915855
C	5.475862	0.665964	1.711892
C	6.232640	0.378854	0.425641
C	4.023882	-0.410825	3.257139
C	2.964030	-1.494648	3.350350
H	-3.360191	4.742198	-1.420930
H	-5.391128	3.061337	-0.717680
H	-0.405476	4.306710	-1.213367
H	-0.544560	3.207797	-2.580736
H	0.301288	1.137366	-3.466995
H	1.920776	0.470871	-3.728454
H	0.862293	-0.240355	-2.494843
H	3.091151	2.641346	-3.332510
H	1.520365	3.436857	-3.195032
H	2.735766	3.647075	-1.921084
H	2.648397	0.066923	-0.663954
H	3.671379	0.613190	-1.994898

H	3.503402	1.613956	-0.549010
H	-0.767873	4.141995	1.109065
H	-1.037920	2.518570	1.753311
H	-0.022062	3.669192	2.645002
H	2.506193	1.255769	1.410015
H	1.882767	2.160237	2.794922
H	0.892982	0.904474	2.035132
H	2.157891	4.438948	1.711255
H	2.930343	3.623720	0.345804
H	1.568776	4.730050	0.067488
H	-4.838999	-0.202904	-1.133172
H	-5.185592	0.303314	0.510180
H	-1.648097	-1.857722	-2.028244
H	-2.786653	-3.048944	-2.685366
H	-3.276128	-1.354448	-2.519904
H	-4.700098	-3.906605	-1.320737
H	-5.107980	-3.210984	0.252391
H	-5.330586	-2.260532	-1.228759
H	-1.319485	-3.278475	0.145515
H	-2.756807	-3.954125	0.935755
H	-2.329757	-4.459227	-0.704177
H	-4.622120	-1.798775	3.687064
H	-5.394457	-1.423486	2.138505
H	-4.410501	-2.892819	2.313052
H	-1.835735	-2.740408	2.497620
H	-1.071494	-1.145640	2.597046
H	-2.094661	-1.727151	3.921781
H	-3.368900	0.352607	3.854389
H	-2.432291	0.976027	2.484880

H	-4.200771	0.930119	2.402901
H	2.922212	-3.820399	-2.955298
H	2.352419	-4.707418	-1.523494
H	3.864267	-1.982979	0.154701
H	3.964524	-3.731720	-0.142977
H	3.605670	0.565175	3.547637
H	4.861523	-0.625113	3.940524
H	4.975143	1.647318	1.667932
H	6.174572	0.692330	2.562375
H	1.486735	-2.176192	-0.219133
H	2.593090	-1.573838	4.378081
H	2.121746	-1.268958	2.689479
H	3.381144	-2.464171	3.056129
H	6.966847	1.168885	0.233543
H	6.760936	-0.577376	0.501222
H	5.549642	0.325174	-0.426515
H	0.528140	-4.422498	-3.244888
H	0.106825	-3.607554	-1.728550
H	0.687085	-2.658905	-3.115818
H	5.967318	-2.472014	-1.038620
H	5.101384	-3.286427	-2.352282
H	4.893138	-1.537644	-2.097536

Supplementary Table 18 | Cartesian coordinates of optimized intermediates and transition states of IV.

SCF energy = -1834.67289673 hartree

ZPE = 0.612749 hartree

Atom	Coordinates (Angstroms)		
	X	Y	Z
Fe	0.003276	-0.551852	-0.154747
P	2.325778	-0.023310	-0.034415
P	-2.329773	-0.047880	-0.053778
N	-0.008410	1.046442	-1.402158
N	0.026810	-2.529373	0.042372
N	0.036119	-3.536266	0.549568
C	1.087891	1.856631	-1.620472
C	0.670623	3.111978	-2.034091
C	-0.755994	3.074435	-2.076498
C	-1.130761	1.798784	-1.686479
C	2.456165	1.287645	-1.377436
C	3.621292	-1.319969	-0.564190
C	2.995206	-2.050366	-1.775113
C	4.981300	-0.738190	-0.998500
C	3.832437	-2.343860	0.567256
C	2.835629	0.872394	1.570521
C	1.978350	2.154549	1.629492
C	2.433996	-0.017174	2.765909
C	4.320705	1.252530	1.675728
C	-2.478898	1.171678	-1.475586
C	-3.623038	-1.387647	-0.467343

C	-3.024717	-2.194541	-1.643364
C	-4.996999	-0.844304	-0.906549
C	-3.788489	-2.333808	0.737018
C	-2.818778	0.951145	1.496672
C	-4.319102	1.234360	1.670545
C	-2.278551	0.188610	2.724844
C	-2.073472	2.301651	1.404538
H	1.306988	3.954330	-2.280422
H	-1.420217	3.885112	-2.353655
H	3.186308	2.061105	-1.112606
H	2.847921	0.786969	-2.272946
H	2.799770	-1.372169	-2.612756
H	3.688796	-2.823902	-2.130556
H	2.051868	-2.536375	-1.512473
H	5.622491	-1.553278	-1.361250
H	4.879177	-0.017758	-1.816135
H	5.510395	-0.246792	-0.180090
H	2.882000	-2.749765	0.930940
H	4.433020	-3.184129	0.194435
H	4.370686	-1.910501	1.416016
H	2.213981	2.862991	0.830892
H	0.912461	1.922163	1.551691
H	2.144057	2.656683	2.591943
H	2.995490	-0.954559	2.802591
H	2.621055	0.520088	3.705306
H	1.366382	-0.266921	2.728445
H	4.479048	1.873680	2.567726
H	4.961979	0.371727	1.774751
H	4.658989	1.829802	0.808363

H	-2.803447	0.594502	-2.351606
H	-3.258054	1.918350	-1.285099
H	-2.066976	-2.649431	-1.378909
H	-3.718309	-2.999803	-1.919567
H	-2.868173	-1.576063	-2.533852
H	-5.635902	-1.683421	-1.214273
H	-5.517990	-0.314885	-0.107124
H	-4.913351	-0.168215	-1.763726
H	-2.822990	-2.718533	1.084125
H	-4.287629	-1.845734	1.579812
H	-4.404974	-3.195070	0.446912
H	-4.466387	1.903343	2.529331
H	-4.743023	1.731633	0.791192
H	-4.894936	0.325045	1.865090
H	-2.739184	-0.796306	2.846023
H	-1.193946	0.041639	2.647130
H	-2.476484	0.765635	3.638002
H	-2.150571	2.820813	2.368999
H	-1.014060	2.173174	1.168272
H	-2.491890	2.956027	0.635484

Supplementary Table 19 | Cartesian coordinates of optimized intermediates and transition states of V-H$^+$(OEt$_2$)$_2$.

SCF energy = -3369.80766784 hartree

ZPE = 1.185238 hartree

Atom	Coordinates (Angstroms)		
	X	Y	Z
N	1.718529	0.265463	-1.898799
C	2.289254	-0.805153	-2.560187
C	3.378808	-0.364099	-3.304780
C	3.486164	1.043757	-3.085505
C	2.453744	1.393693	-2.221476
C	1.745615	-2.185229	-2.301502
P	0.163797	-2.005122	-1.292042
C	0.320567	-3.419924	-0.011001
C	-1.029758	-3.619662	0.700592
C	2.027263	2.711719	-1.630426
P	0.273042	2.458356	-1.006531
C	0.122874	3.678355	0.451855
C	-1.356257	3.779657	0.875972
Fe	0.143939	0.199951	-0.717016
N	-0.881506	0.114040	0.705636
N	-1.467320	0.051456	1.721783
C	-0.778345	3.053099	-2.502732
C	-0.830171	4.573303	-2.722212
C	-2.203530	2.502637	-2.310357
C	-0.180247	2.404149	-3.771715
C	-1.231613	-2.402229	-2.546475

C	-1.278322	-3.849952	-3.057271
C	-2.584456	-2.021150	-1.909114
C	-1.008688	-1.448816	-3.739692
C	0.912821	3.032221	1.610721
C	0.703002	5.083850	0.203789
C	0.824281	-4.768548	-0.560863
C	1.338037	-2.916134	1.034079
K	3.176641	0.012389	0.524602
O	4.777370	-2.116928	1.105533
C	5.886601	-2.593498	0.332144
C	5.898224	-1.871326	-1.003773
C	4.752190	-2.610841	2.455097
C	5.485228	-1.675912	3.410870
O	3.478938	1.292889	2.932444
C	4.340683	2.423495	3.089962
C	5.184044	2.584652	1.835751
C	2.620628	1.092817	4.062165
C	1.751484	-0.129960	3.824326
H	4.027820	-0.976803	-3.920448
H	4.217514	1.716612	-3.519851
H	2.455532	-2.779334	-1.711334
H	1.575976	-2.753596	-3.223599
H	-0.069975	-1.638374	-4.269211
H	-1.828993	-1.573045	-4.459653
H	-1.003530	-0.407496	-3.407065
H	-2.018687	-3.927774	-3.865442
H	-0.312969	-4.174970	-3.461140
H	-1.576719	-4.550636	-2.272225
H	-2.551870	-0.990706	-1.541314

H	-3.379629	-2.097462	-2.663701
H	-2.856347	-2.671030	-1.073084
H	2.336783	-2.781730	0.602182
H	1.007294	-1.974007	1.482700
H	1.436964	-3.659451	1.837106
H	-1.767521	-4.097571	0.049270
H	-0.890134	-4.272850	1.572939
H	-1.437017	-2.668772	1.053883
H	0.929149	-5.475815	0.273412
H	0.137416	-5.211920	-1.283108
H	1.806296	-4.678913	-1.036530
H	2.100115	3.537057	-2.348393
H	2.645783	3.006747	-0.770400
H	-0.015678	1.332380	-3.647363
H	-0.875219	2.554420	-4.608680
H	0.776365	2.854241	-4.051945
H	-1.362608	4.784676	-3.660048
H	-1.364413	5.090678	-1.920340
H	0.171767	5.008061	-2.807998
H	-2.172742	1.422110	-2.132713
H	-2.708982	2.968522	-1.460179
H	-2.806987	2.699373	-3.207159
H	0.672912	5.654811	1.142150
H	1.749014	5.040376	-0.119009
H	0.141544	5.648785	-0.541334
H	-1.961673	4.305556	0.131322
H	-1.790025	2.788452	1.043132
H	-1.427928	4.343766	1.815971
H	0.803097	3.648544	2.513683

H	0.551688	2.026052	1.833421
H	1.984428	2.970702	1.393855
H	6.825502	-2.416145	0.874778
H	5.781054	-3.678899	0.187741
H	3.694787	-2.691391	2.728547
H	5.175756	-3.623731	2.481647
H	1.998505	1.988009	4.206197
H	3.238531	0.960187	4.962984
H	3.733098	3.324048	3.263264
H	4.981999	2.273380	3.971286
H	1.123458	-0.312992	4.702772
H	1.084296	0.008394	2.966482
H	2.369599	-1.020049	3.661516
H	5.873930	3.426761	1.952518
H	5.781064	1.684126	1.647050
H	4.556004	2.792026	0.959859
H	6.695549	-2.271273	-1.639379
H	4.946131	-1.994447	-1.531678
H	6.081976	-0.798076	-0.871864
H	5.439920	-2.069396	4.433192
H	6.539793	-1.574502	3.131205
H	5.022609	-0.683022	3.399462
O	-5.191054	0.862952	1.233416
H	-5.563146	-0.143286	1.302118
O	-6.180616	-1.404768	1.444528
C	-4.337866	1.075016	0.038982
C	-4.603670	1.323097	2.516964
C	-7.435704	-1.519880	0.727546
C	-5.303032	-2.548727	1.293955

C	-5.069255	0.545475	-1.175048
H	-4.194037	2.155112	-0.002965
H	-3.379381	0.586705	0.221048
C	-5.591703	1.033048	3.625206
H	-3.645100	0.813346	2.634331
H	-4.436027	2.392759	2.376591
C	-8.279390	-0.291914	1.016769
H	-7.225185	-1.624985	-0.344277
H	-7.928669	-2.434056	1.076395
C	-4.120031	-2.377391	2.225942
H	-5.886148	-3.441412	1.546232
H	-4.989520	-2.623271	0.244091
H	-4.496624	0.809698	-2.067878
H	-5.155435	-0.543982	-1.145528
H	-6.067205	0.986655	-1.256491
H	-9.245700	-0.388512	0.512030
H	-8.456487	-0.191711	2.092290
H	-7.791883	0.618935	0.655880
H	-3.497782	-3.276520	2.190304
H	-3.492535	-1.527831	1.938453
H	-4.462553	-2.235293	3.256218
H	-5.172024	1.398691	4.567726
H	-6.543586	1.542123	3.446230
H	-5.775408	-0.040493	3.722380

Supplementary Table 20 | Cartesian coordinates of optimized intermediates and transition states of TS_{VI}.

SCF energy = -3369.80242965 hartree

ZPE = 1.186978 hartree

Imaginary frequency: 165i cm^{-1}

Atom	X	Y	Z
C	-2.433543	-1.584613	-1.826621
N	-1.652187	-0.444801	-1.745779
C	-2.354405	0.585943	-2.345023
C	-3.577245	0.107348	-2.807195
C	-3.628122	-1.283383	-2.474374
Fe	0.167184	-0.335312	-1.021178
N	1.754189	-0.228630	-0.299065
N	2.852275	-0.186955	0.115256
C	-1.746235	1.963603	-2.340930
P	0.051934	1.776854	-1.821905
C	1.019154	1.991055	-3.459251
C	0.397663	0.983377	-4.450937
C	-1.918782	-2.851509	-1.196362
P	-0.098033	-2.577678	-0.817538
C	0.800619	-3.538939	-2.207089
C	0.161672	-3.070838	-3.533025
C	0.328404	3.278170	-0.672289
C	-0.373837	2.899060	0.650037
C	1.836357	3.444056	-0.401322
C	-0.257950	4.617490	-1.158571

C	0.132355	-3.446097	0.868563
C	-0.545131	-4.821548	1.013834
C	1.636059	-3.567389	1.182840
C	-0.501023	-2.480203	1.891627
C	0.709827	-5.070505	-2.123786
C	2.278850	-3.095990	-2.217376
C	0.972974	3.397736	-4.077026
C	2.484448	1.567439	-3.224301
K	-3.028540	0.181529	0.688249
O	-3.848194	-1.629473	2.564824
C	-3.308968	-1.693072	3.890682
C	-2.535990	-0.415973	4.177723
C	-4.528034	-2.834150	2.186332
C	-5.290233	-2.596771	0.893162
O	-3.783661	2.690715	1.369959
C	-3.281713	3.274588	2.583770
C	-4.362143	3.381044	3.652785
C	-4.375755	3.639239	0.468832
C	-5.173574	2.888207	-0.583601
O	3.846267	1.255363	2.730003
C	2.420551	1.303381	2.883496
C	1.931036	-0.044663	3.378382
C	4.448742	2.544870	2.558539
C	5.925182	2.446388	2.908036
O	5.398516	-0.241307	1.039218
C	5.363646	-1.669915	1.469827
C	5.373210	-1.715528	2.980441
C	5.759015	-0.029113	-0.395916
C	5.599773	1.434526	-0.731642

H	-4.336935	0.679575	-3.327537
H	-4.433940	-1.975109	-2.692662
H	-2.239522	2.618113	-1.610740
H	-1.845525	2.469318	-3.307735
H	-0.632513	1.236176	-4.720292
H	0.991628	0.973859	-5.374439
H	0.399626	-0.026714	-4.032284
H	1.432382	3.371762	-5.074701
H	-0.053473	3.763006	-4.195249
H	1.531656	4.124901	-3.480293
H	2.532843	0.564589	-2.788388
H	3.017421	1.559355	-4.184976
H	3.015638	2.251344	-2.557418
H	-1.461065	2.849303	0.528737
H	-0.025569	1.929132	1.020539
H	-0.169098	3.663776	1.412010
H	2.362726	3.835994	-1.276749
H	1.985672	4.158356	0.419918
H	2.301716	2.496876	-0.113963
H	-0.098534	5.380338	-0.383728
H	0.215278	4.976005	-2.074009
H	-1.337454	4.555372	-1.334571
H	-2.081235	-3.732702	-1.826940
H	-2.426611	-3.066380	-0.247205
H	0.133279	-1.979562	-3.594449
H	0.758647	-3.448845	-4.373506
H	-0.860247	-3.441362	-3.659355
H	1.140101	-5.509399	-3.034571
H	1.270396	-5.469485	-1.273266

H	-0.326844	-5.417711	-2.049251
H	2.351660	-2.008516	-2.319228
H	2.807618	-3.384452	-1.304491
H	2.794404	-3.565788	-3.066161
H	-0.415909	-5.176893	2.045654
H	-1.622289	-4.771119	0.820145
H	-0.115975	-5.574394	0.350921
H	2.124204	-4.321109	0.557781
H	2.149960	-2.612216	1.039532
H	1.767419	-3.871976	2.230176
H	-0.331215	-2.854057	2.910878
H	-0.066500	-1.479887	1.806666
H	-1.584371	-2.396882	1.754651
H	-5.030323	4.322422	1.024659
H	-3.574725	4.235633	0.005928
H	-2.471216	2.619946	2.923051
H	-2.837851	4.254497	2.362443
H	-2.650800	-2.569372	3.977388
H	-4.133062	-1.816478	4.608511
H	-3.789817	-3.642125	2.066679
H	-5.220034	-3.125857	2.989262
H	-2.152896	-0.434728	5.203400
H	-1.675685	-0.313110	3.505034
H	-3.183041	0.462878	4.075473
H	-5.826787	-3.507637	0.607037
H	-6.024730	-1.792916	1.019456
H	-4.621448	-2.335915	0.064521
H	-5.629384	3.598356	-1.281990
H	-4.539899	2.209323	-1.165642

H	-5.973934	2.304211	-0.114924
H	-3.940503	3.801853	4.572899
H	-5.182997	4.028998	3.328295
H	-4.772818	2.390790	3.879629
H	4.490778	0.166784	1.244250
H	6.794656	-0.366679	-0.454031
H	5.098609	-0.667492	-0.983197
H	4.467766	-2.103102	1.020595
H	6.257705	-2.112378	1.029546
H	4.299938	2.891804	1.526134
H	3.952472	3.266515	3.222772
H	2.169777	2.092435	3.608202
H	1.954229	1.552946	1.926110
H	5.906611	1.577090	-1.773116
H	4.554783	1.739116	-0.640793
H	6.227523	2.063067	-0.094530
H	6.409516	3.419095	2.769184
H	6.047184	2.141677	3.952725
H	6.431969	1.712147	2.276430
H	0.847102	-0.011669	3.532959
H	2.144791	-0.820054	2.639546
H	2.409509	-0.306675	4.328458
H	5.316169	-2.763585	3.292741
H	6.294419	-1.279733	3.377369
H	4.519453	-1.176869	3.395092

Supplementary Table 21 | Cartesian coordinates of optimized intermediates and transition states of VI-2Et_2O.

SCF energy = -3369.84694382 hartree

ZPE = 1.185368 hartree

Atom	Coordinates (Angstroms)		
	X	Y	Z
C	-3.268385	-2.588051	-1.987907
C	-2.121476	-2.485757	-1.214635
N	-1.448908	-1.316846	-1.548285
C	-2.181124	-0.677011	-2.539125
C	-3.305081	-1.433672	-2.835244
C	-1.538656	-3.363085	-0.139802
P	0.239548	-2.826541	0.097367
C	0.589302	-3.080840	1.944539
C	-0.131424	-1.927101	2.674947
Fe	0.237669	-0.727228	-0.823368
P	-0.018398	1.004430	-2.295505
C	-0.137676	2.804288	-1.686750
C	-0.811247	3.769736	-2.682786
C	-1.710489	0.664129	-3.035685
N	1.288763	0.044572	0.245331
N	1.777941	0.695061	1.162941
C	1.210137	0.809002	-3.735443
C	2.645172	0.796727	-3.166619
C	0.940159	-0.586834	-4.339907
C	1.080663	1.876847	-4.833061
C	1.239701	-4.021498	-1.002723

C	2.634364	-3.401415	-1.221443
C	0.521962	-4.094701	-2.369046
C	1.374941	-5.448848	-0.447584
C	-0.990882	2.750345	-0.400565
C	1.257860	3.343591	-1.323703
C	0.063681	-4.416277	2.507460
C	2.104341	-2.954711	2.201347
O	4.534371	-0.411062	1.428404
C	5.782638	-0.662979	0.785924
C	5.520034	-0.898929	-0.692210
C	4.609075	-0.264961	2.850004
C	5.217390	1.063977	3.291766
K	-3.021453	-0.008054	0.488870
O	-3.055860	-0.199947	3.189473
C	-3.806015	-1.233592	3.838335
C	-4.670880	-1.946157	2.811480
C	-2.215501	0.521828	4.102696
C	-1.424473	1.573571	3.345279
O	-4.503046	2.227512	0.111349
C	-5.644788	2.376813	-0.745569
C	-5.728327	1.174307	-1.668973
C	-4.415649	3.231509	1.138678
C	-5.115759	2.789815	2.419236
O	3.430082	3.400640	1.329724
C	4.280480	3.980456	0.347951
C	4.994292	2.857052	-0.384023
C	2.647730	4.358305	2.033235
C	1.829151	3.629363	3.086091
H	-4.067195	-1.191485	-3.566099

H	-3.989877	-3.396375	-1.962352
H	-2.391126	1.462668	-2.718190
H	-1.674641	0.718399	-4.128999
H	-0.057766	-0.678708	-4.779482
H	1.673501	-0.778552	-5.133228
H	1.053611	-1.370065	-3.584613
H	1.729776	1.606751	-5.676324
H	0.055906	1.953807	-5.213213
H	1.394169	2.863747	-4.481280
H	2.750679	0.049773	-2.372717
H	3.347047	0.544690	-3.972115
H	2.943752	1.764773	-2.759431
H	-2.030381	2.472962	-0.615094
H	-0.562245	2.058696	0.331234
H	-1.020637	3.748167	0.055131
H	1.884828	3.477632	-2.209608
H	1.141624	4.329611	-0.855898
H	1.782436	2.704500	-0.610104
H	-0.893980	4.757035	-2.209572
H	-0.234607	3.892781	-3.601539
H	-1.823601	3.453649	-2.953606
H	-1.612658	-4.428316	-0.381863
H	-2.051544	-3.236504	0.822426
H	0.317678	-3.103926	-2.781566
H	1.167084	-4.631852	-3.075802
H	-0.427736	-4.634781	-2.310080
H	1.865630	-6.077874	-1.201979
H	1.987016	-5.486604	0.457752
H	0.398949	-5.895832	-0.228853

H	2.555477	-2.401819	-1.663131
H	3.198320	-3.309170	-0.290129
H	3.211988	-4.036529	-1.905570
H	0.263455	-4.445899	3.586676
H	-1.018528	-4.521201	2.372308
H	0.548867	-5.284571	2.059582
H	2.653842	-3.824283	1.828525
H	2.529614	-2.057330	1.743445
H	2.279221	-2.895453	3.283426
H	0.047850	-2.023444	3.753840
H	0.240375	-0.952207	2.356371
H	-1.216788	-1.950334	2.529479
H	-6.557174	2.461453	-0.140057
H	-5.536422	3.305347	-1.324740
H	-3.346684	3.393132	1.314163
H	-4.831254	4.175485	0.762645
H	-1.536111	-0.184635	4.599910
H	-2.845030	0.988714	4.874144
H	-3.111248	-1.940975	4.314432
H	-4.430593	-0.789710	4.626843
H	-0.791311	2.131852	4.042063
H	-0.764006	1.120375	2.599111
H	-2.089685	2.290108	2.851835
H	-5.267980	-2.722716	3.300028
H	-5.364150	-1.247549	2.326895
H	-4.058079	-2.438828	2.045548
H	-6.559852	1.298486	-2.370740
H	-4.806663	1.056370	-2.248818
H	-5.902803	0.251023	-1.103117

H	-5.031381	3.570995	3.183574
H	-6.179972	2.601280	2.240195
H	-4.660143	1.873583	2.811934
H	2.812363	0.735061	1.083839
H	6.465641	0.187782	0.919116
H	6.259507	-1.544626	1.243760
H	3.572284	-0.335194	3.193610
H	5.163535	-1.115520	3.276836
H	3.683675	4.582691	-0.355901
H	5.004417	4.658456	0.829448
H	3.303603	5.113091	2.497680
H	1.987242	4.888357	1.327285
H	6.463907	-1.071920	-1.220847
H	4.873881	-1.769391	-0.834888
H	5.026165	-0.029349	-1.136678
H	5.659184	3.262568	-1.154402
H	5.587978	2.260719	0.314554
H	4.266829	2.194252	-0.862221
H	1.174540	4.332951	3.612934
H	1.221036	2.852074	2.615442
H	2.486196	3.148806	3.819373
H	5.147256	1.158449	4.382068
H	6.275589	1.139234	3.018852
H	4.676892	1.895731	2.829111

Supplementary Table 22 | Cartesian coordinates of optimized intermediates and transition states of TS_{VII}.

SCF energy = -3369.78970118 hartree

ZPE = 1.187283 hartree

Imaginary frequency: 162i cm^{-1}

Atom	X	Y	Z
C	1.297615	0.251973	-2.317987
C	0.407671	-0.658361	-1.749253
N	0.429282	-0.528125	-0.371427
C	1.345492	0.451932	-0.056350
C	1.906242	0.962848	-1.232608
C	-0.554725	-1.642148	-2.362907
P	-1.411955	-2.540217	-0.939920
C	-3.203912	-2.746727	-1.575520
C	-3.848832	-1.354301	-1.423442
Fe	-0.901805	-1.279727	0.855714
P	0.279462	-0.167612	2.406675
C	-0.519212	1.134211	3.572224
C	0.442168	2.167035	4.194310
C	1.488692	0.869778	1.385581
N	-2.311541	-1.582888	1.792080
N	-3.309461	-1.675748	2.364432
C	1.411340	-1.323839	3.441718
C	0.526220	-2.418919	4.074343
C	2.349343	-2.021501	2.436479
C	2.271481	-0.643564	4.518333

C	-0.539425	-4.250265	-0.891889
C	-0.892879	-4.917367	0.454076
C	0.975337	-3.964863	-0.888279
C	-0.853140	-5.200330	-2.056917
C	-1.540973	1.900476	2.707563
C	-1.293143	0.413589	4.693679
C	-3.346537	-3.182246	-3.047527
C	-3.964711	-3.731320	-0.665944
K	-1.700191	1.530872	-0.625547
O	-4.332469	1.859057	-1.369124
C	-4.715411	1.954543	-2.743243
C	-3.471839	2.142962	-3.597119
C	-5.457048	1.694811	-0.495029
C	-4.971660	1.594577	0.940521
O	-1.311214	4.214744	0.010746
C	-0.361999	5.119696	-0.566923
C	0.843529	4.327101	-1.040384
C	-2.460670	4.864229	0.580990
C	-3.543217	5.153812	-0.453651
C	3.674511	-1.856690	-1.129062
O	4.534488	-0.644573	-0.937058
C	5.343997	-0.541684	0.319658
C	6.769872	-0.215006	-0.051440
C	4.551052	-3.015376	-1.537020
O	4.900783	2.018523	-1.620957
C	4.895895	2.169032	-3.045821
C	4.917203	0.798998	-3.702343
C	4.818438	3.277479	-0.944150
C	4.778457	3.044507	0.555801

H	2.571846	1.809450	-1.311750
H	1.489517	0.393121	-3.375913
H	1.228215	1.927649	1.507927
H	2.514276	0.770905	1.763002
H	3.045633	-1.319218	1.967591
H	2.946458	-2.779848	2.960526
H	1.774302	-2.520945	1.652370
H	2.968602	-1.378375	4.944765
H	2.867990	0.180208	4.108458
H	1.668393	-0.254634	5.342792
H	-0.093583	-2.896587	3.307665
H	1.165393	-3.183172	4.537379
H	-0.138594	-2.029812	4.848905
H	-1.051065	2.490606	1.923410
H	-2.265459	1.215029	2.257559
H	-2.092796	2.612085	3.336407
H	-0.619930	-0.047990	5.421226
H	-1.908467	1.144748	5.235161
H	-1.958636	-0.357837	4.295884
H	-0.143604	2.888359	4.780599
H	1.171306	1.715885	4.868534
H	0.987682	2.737135	3.434213
H	-0.068612	-2.336885	-3.058647
H	-1.327136	-1.127059	-2.947966
H	1.229125	-3.246122	-0.106739
H	1.521962	-4.897580	-0.694510
H	1.325996	-3.566959	-1.846269
H	-0.208007	-6.087162	-1.985324
H	-1.888525	-5.550411	-2.037995

H	-0.663464	-4.730737	-3.028839
H	-0.654694	-4.245112	1.286227
H	-1.952666	-5.174638	0.526831
H	-0.313530	-5.843988	0.568453
H	-4.410406	-3.164933	-3.321984
H	-2.823499	-2.504180	-3.730767
H	-2.980707	-4.194142	-3.228293
H	-3.625634	-4.761677	-0.805035
H	-3.859204	-3.470004	0.391098
H	-5.033921	-3.701992	-0.915994
H	-4.907120	-1.402364	-1.712962
H	-3.791113	-0.999209	-0.392113
H	-3.375642	-0.610784	-2.074283
H	-0.818964	5.666542	-1.403356
H	-0.062529	5.857290	0.192376
H	-2.835858	4.179685	1.348674
H	-2.140163	5.783933	1.089538
H	-6.008592	0.787700	-0.781004
H	-6.131933	2.555368	-0.616541
H	-5.250631	1.039918	-3.037757
H	-5.401551	2.804967	-2.872098
H	-5.830495	1.536389	1.617498
H	-4.365131	0.697104	1.102834
H	-4.385863	2.478652	1.215767
H	-3.753265	2.252449	-4.649499
H	-2.924675	3.045313	-3.298342
H	-2.803823	1.274992	-3.524573
H	1.603495	5.005066	-1.444278
H	1.285141	3.764598	-0.211627

H	0.574779	3.614683	-1.827903
H	-4.402375	5.632252	0.031011
H	-3.178076	5.827976	-1.235975
H	-3.882253	4.222540	-0.918783
H	4.016647	0.197090	-1.099341
H	3.136079	-2.009882	-0.197381
H	2.972814	-1.547661	-1.901542
H	4.859667	0.226955	0.920565
H	5.235123	-1.507865	0.809661
H	3.995401	2.725290	-3.349322
H	5.771613	2.761722	-3.349137
H	5.686761	3.893834	-1.220142
H	3.913239	3.812134	-1.272665
H	3.904013	-3.880840	-1.712534
H	5.092060	-2.789039	-2.460256
H	5.268704	-3.286827	-0.756305
H	4.971287	0.913712	-4.789942
H	5.784425	0.219622	-3.370588
H	4.007139	0.238795	-3.463211
H	4.731643	4.005601	1.078515
H	3.894552	2.461562	0.836648
H	5.675544	2.514927	0.892640
H	7.355569	-0.107054	0.867978
H	7.208041	-1.017522	-0.651909
H	6.807692	0.721821	-0.611045

Supplementary Table 23 | Cartesian coordinates of optimized intermediates and transition states of VII-2Et$_2$O.

SCF energy = -3369.86260248 hartree

ZPE = 1.184598 hartree

Atom	Coordinates (Angstroms)		
	X	Y	Z
C	1.342700	-1.360980	1.650975
C	0.414771	-1.418241	0.662456
N	-0.598087	-0.452160	0.811655
C	-0.252079	0.339505	1.906203
C	0.922799	-0.285911	2.631534
C	0.308998	-2.334378	-0.521460
P	-1.498551	-2.433518	-0.980308
C	-1.482224	-2.726149	-2.857440
C	-1.212874	-1.334800	-3.469529
Fe	-2.310759	-0.496516	-0.083473
P	-2.816212	1.163772	1.408459
C	-2.874771	2.933081	0.719186
C	-3.214949	4.017537	1.753761
C	-1.340807	1.127386	2.567903
N	-3.811441	-0.438374	-1.028637
N	-4.767064	-0.404910	-1.647820
C	-4.296476	0.774387	2.538832
C	-5.619328	1.047482	1.798629
C	-4.191482	-0.743438	2.815310
C	-4.294958	1.531726	3.880582
C	-2.161170	-3.937238	-0.019740

C	-3.702687	-3.931170	-0.090544
C	-1.751678	-3.722193	1.455459
C	-1.615276	-5.291606	-0.501045
C	-1.469729	3.199158	0.142429
C	-3.870400	2.990599	-0.457853
C	-0.384657	-3.700914	-3.329379
C	-2.858040	-3.217312	-3.346667
K	1.514994	1.360730	-0.407347
O	1.664608	0.586783	-3.144462
C	2.526760	-0.372024	-3.771956
C	3.427376	-1.003745	-2.726743
C	0.932828	1.360671	-4.107122
C	-0.085738	2.223885	-3.384451
O	2.062837	4.073742	-1.196652
C	3.200885	4.308508	-2.038028
C	4.021203	3.032216	-2.124867
C	1.359661	5.269306	-0.827231
C	0.528491	5.868665	-1.956941
C	4.637641	-4.120365	1.171456
O	4.235860	-2.971086	1.910305
C	4.576698	-2.989060	3.295301
C	6.032364	-2.606770	3.554799
C	4.138987	-3.955393	-0.256078
O	3.579452	1.679840	1.560921
C	4.595763	0.701325	1.807365
C	4.852294	-0.079553	0.533368
C	3.429441	2.594940	2.649734
C	2.248232	3.506247	2.366289
H	1.728734	0.420002	2.879776

H	2.212401	-1.997697	1.753031
H	-1.039161	2.140436	2.863289
H	-1.656806	0.628073	3.498357
H	-3.255725	-1.010940	3.319260
H	-5.020581	-1.053542	3.464112
H	-4.251858	-1.322399	1.886496
H	-5.153149	1.196287	4.477581
H	-3.392930	1.334887	4.468519
H	-4.387183	2.611601	3.748342
H	-5.645017	0.572949	0.813024
H	-6.449942	0.639756	2.389067
H	-5.800351	2.119269	1.674974
H	-0.701907	3.241898	0.921982
H	-1.201986	2.426106	-0.588250
H	-1.469972	4.162168	-0.381174
H	-4.900909	2.808030	-0.146446
H	-3.831184	3.989623	-0.911260
H	-3.613289	2.256920	-1.228025
H	-3.080056	5.007917	1.299115
H	-4.255056	3.947482	2.084141
H	-2.567949	3.964435	2.636394
H	0.762104	-3.311161	-0.328428
H	0.822132	-1.908801	-1.390795
H	-2.082710	-2.746487	1.827403
H	-2.221543	-4.499586	2.071329
H	-0.669284	-3.787560	1.600161
H	-1.961148	-6.081222	0.178980
H	-1.970040	-5.543682	-1.504233
H	-0.519948	-5.314712	-0.504178

H	-4.111498	-2.977568	0.260180
H	-4.078500	-4.106766	-1.100945
H	-4.095398	-4.727304	0.554838
H	-0.424822	-3.777302	-4.424057
H	0.619682	-3.353786	-3.064116
H	-0.515228	-4.706578	-2.923889
H	-3.059857	-4.246332	-3.036625
H	-3.671318	-2.579130	-2.987230
H	-2.873889	-3.194828	-4.443961
H	-1.151785	-1.422912	-4.562372
H	-2.015711	-0.631003	-3.227960
H	-0.271277	-0.903804	-3.117124
H	2.872998	4.622448	-3.037933
H	3.801615	5.124213	-1.609317
H	0.712576	4.978350	0.003689
H	2.082005	6.006293	-0.445498
H	0.433754	0.682942	-4.813175
H	1.639825	1.982664	-4.676424
H	1.910317	-1.132209	-4.274302
H	3.130490	0.132087	-4.540679
H	-0.650847	2.820323	-4.108609
H	-0.798887	1.602905	-2.830142
H	0.410664	2.915494	-2.696426
H	4.055847	-1.771148	-3.190825
H	4.083649	-0.255192	-2.273023
H	2.845773	-1.484683	-1.933569
H	4.907639	3.198801	-2.746252
H	4.353598	2.722393	-1.127533
H	3.437452	2.223412	-2.577676

H	-0.004634	6.752519	-1.588315
H	1.152576	6.181196	-2.800256
H	-0.211079	5.147876	-2.320366
H	0.605299	-0.717453	3.608007
H	5.732154	-4.229562	1.182796
H	4.210802	-5.027075	1.630301
H	3.907005	-2.260145	3.765272
H	4.350385	-3.976828	3.726275
H	5.513341	1.207618	2.142946
H	4.267633	0.027452	2.612723
H	3.270490	2.035749	3.584677
H	4.355510	3.178857	2.764119
H	4.426411	-4.820735	-0.863011
H	3.047382	-3.864290	-0.272838
H	4.564033	-3.053736	-0.709053
H	5.625386	-0.833124	0.706127
H	3.949662	-0.612802	0.221260
H	5.179948	0.589066	-0.269867
H	2.145413	4.253099	3.160579
H	2.391422	4.025774	1.414187
H	1.317385	2.927883	2.320752
H	6.229230	-2.580596	4.632775
H	6.725685	-3.324272	3.103227
H	6.242962	-1.614990	3.139465

Supplementary Methods.

General.

^{1}H NMR (270 MHz), $^{31}P\{^1H\}$ NMR (109 MHz), and $^{15}N\{^1H\}$ NMR (27 MHz) spectra were recorded on a JEOL Excalibur 270 spectrometer in suitable solvent, and spectra were referenced to residual solvent (^{1}H) or external standard ($^{31}P\{^1H\}$: H_3PO_4, $^{15}N\{^1H\}$: CH_3NO_2). IR spectra were recorded on a JASCO FT/IR 4100 Fourier Transform infrared spectrometer. Magnetic susceptibility was measured in solution by using Evans' method.[1-3] GLC analyses were carried out on a Shimadzu GC-2014 instrument equipped with a flame-ionization detector using a CBP10 fused silica capillary column (25 m x 0.25 mm). Absorption spectra were recorded on a Shimadzu MultiSpec-1500. Evolved dihydrogen and methane were quantified by a gas chromatography using a Shimadzu GC-8A with a TCD detector and a SHINCARBON ST (6 m × 3 mm). X-band EPR spectra were recorded on a JES-FA300 Electron Spin Resonance Spectrometer. Cyclic voltammograms (CVs) were recorded on an ALS/Chi model 610C electrochemical analyzer with platinum working electrode in THF containing 1 mM of sample and 0.1 M of $[N^nBu_4]PF_6$ as a supporting electrolyte at a scan rate of 0.1 V/s at room temperature. All potentials were measured against an $Ag^{0/+}$ electrode and converted to the values vs ferrocene$^{0/+}$. Elemental analyses were performed at Microanalytical Center of The University of Tokyo.

All manipulations were carried out under an atmosphere of nitrogen by using standard Schlenk techniques or glovebox techniques unless otherwise stated. Solvents were dried by general methods, and degassed before use. Sodium metals (30wt% dispersion in toluene, Across Organics) were washed with hexane and then with THF, dried *in vacuo*, and stored in an argon-filled glovebox. Me_3SiCl was distilled before use. 2,5-(di-*tert*-butylphosphinomethyl)-1*H*-pyrrole (PNP-H),[4-6] $[FeCl_2(thf)_{1.5}]$ (thf = tetrahydrofuran),[7] KC_8,[8] $[H(OEt_2)_2]BAr^F_4$ (Ar^F = 3,5-$(CF_3)_2C_6H_3$),[9] and $CoCp^*_2$ (Cp^* =η^5-C_5Me_5)[10] were prepared according to the literature method. All the other reagents were commercially available.

Preparation of PNP-Li.

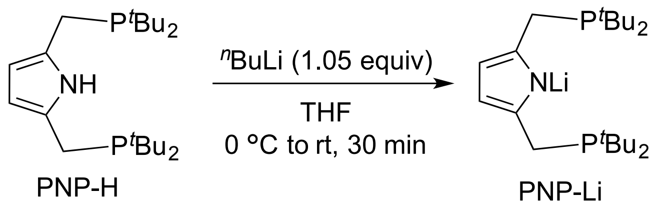

Lithium pyrrolide PNP-Li was prepared from a modified method of the reported procedure.[5,6]

To a solution of PNP-H (1.35 g, 3.52 mmol) in THF (6 mL) was added dropwise ^{n}BuLi (1.55 M in hexane, 2.40 mL, 3.72 mmol) at 0 ºC. The reaction mixture was stirred at room temperature for 30 min, and the solvent was removed and dried *in vacuo*. The obtained solid was washed with pentane (3 mL, 3 times) and dried *in vacuo* to afford PNP-Li as a white solid (1.00 g, 2.57 mmol, 73%). This sample was used for the subsequent reaction without further purification. ^{1}H NMR (THF-d_8) δ 5.47 (s, Ar-*H*, 2H), 2.88 (s, CH_2P^{t}Bu$_2$, 4H), 1.07 (d, $J_{P\text{-}H}$ = 10.0 Hz, CH$_2$P^tBu_2, 36H). ^{31}P{^{1}H} NMR (THF-d_8) δ 21.8 (s).

Preparation of [FeCl(PNP)] (2).

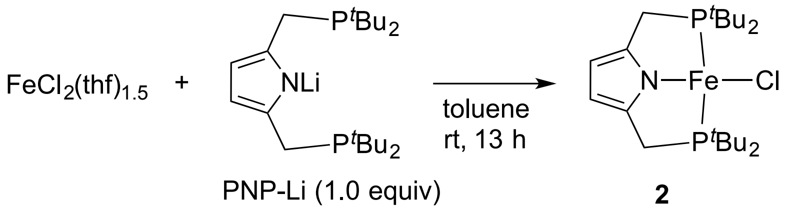

To a suspension of [FeCl$_2$(thf)$_{1.5}$] (476 mg, 2.03 mmol) in toluene (10 mL) was added a suspension of PNP-Li (777 mg, 2.00 mmol) in toluene (20 mL), and then the resultant orange mixture was stirred at room temperature for 13 h. Hexane (10 mL) was added to the orange mixture and stirred at room temperature for several minutes. The mixture was filtered through Celite, and the filter cake was washed with hexane (5 mL, 3 times). After the combined filtrate was concentrated to *ca.* 5 mL, addition of hexane (10 mL) afforded the orange solid **2**, which was collected by decantation, washed with hexane and dried *in vacuo* (808 mg, 1.71 mmol, 85%). Single crystals of **2** suitable for X-ray crystallography were obtained as orange crystals by

recrystallization from hexane at −30 ˚C. ^{1}H NMR (C_6D_6) δ 9.5, −3.7, −25.7. Magnetic susceptibility (Evans' method): μ_{eff} = 3.7±0.2 μ_B in C_6D_6 at 296 K. Anal. Calcd. for $C_{22}H_{42}ClFeNP_2$: C, 55.77; H, 8.93; N, 2.96. Found: C, 55.87; H, 8.78; N, 3.13.

Preparation of [Fe(N_2)(PNP)] (1).

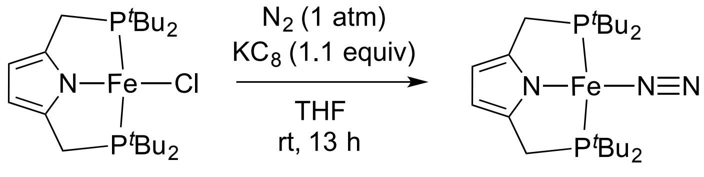

2 **1**

A suspension of **2** (142 mg, 0.300 mmol) and KC_8 (44.4 mg, 0.328 mmol) in THF (6 mL) was stirred at room temperature for 13 h under N_2 (1 atm). The resultant dark red suspension was concentrated *in vacuo*. After the addition of hexane (5 mL) to the dark red residue, the solution was filtered through Celite, and the filter cake was washed with hexane (2 mL, 5 times). The combined filtrate was concentrated to *ca.* 3 mL and the solution was kept at −17 ˚C to give **1** as red crystals, which were collected by decantation, washed with a small amount of cold pentane, and dried *in vacuo* (95.4 mg, 0.205 mmol, 68%). ^{1}H NMR (C_6D_6) δ 3.3, −1.9, −15.1. Magnetic susceptibility (Evans' method): μ_{eff} = 3.0±0.2 μ_B in C_6D_6 at 296 K. IR (KBr, cm^{-1}) 1964 (ν_{NN}). IR (THF, cm^{-1}) 1966 (ν_{NN}). Anal. Calcd. for $C_{22}H_{42}FeN_3P_2$: C, 56.66; H, 9.08; N, 9.01. Found: C, 56.88; H, 9.10; N, 8.58.

Preparation of [FeH(PNP)] (3).

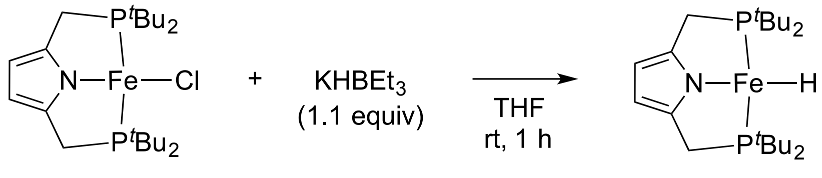

2 **3**

To a solution of **2** (142 mg, 0.300 mmol) in THF (6 mL) was added $KHBEt_3$ (1.0 M in THF, 330 μL, 0.33 mmol) at room temperature. The resultant brown mixture was stirred at room temperature for 1 h, and the solvent was removed *in vacuo*. After the addition of pentane (7 mL) to the brown residue, the solution was filtered through Celite, and the filter cake was washed with

pentane (2 mL, 4 times). The combined filtrate was concentrated to *ca.* 4 mL and the solution was kept at −30 ˚C to give **3** as yellow crystals, which were collected by decantation and dried *in vacuo* (82.0 mg, 0.187 mmol, 62%). ^{1}H NMR (C_6D_6) δ −13.2, −15.0, −15.4. Magnetic susceptibility (Evans' method): μ_{eff} = 3.8±0.2 μ_B in C_6D_6 at 296 K. IR (KBr, cm^{-1}) 1667 (ν_{FeH}). IR (hexane, cm^{-1}) 1684 (ν_{FeH}). Anal. Calcd. for $C_{22}H_{43}FeNP_2$: C, 60.14; H, 9.86; N, 3.19. Found: C, 60.41; H, 9.77; N, 3.46.

Preparation of [FeMe(PNP)] (4).

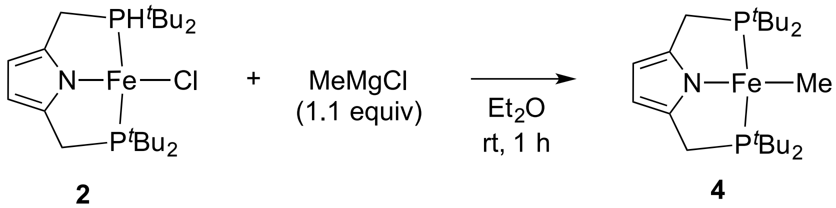

To a solution of **2** (142 mg, 0.300 mmol) in THF (6 mL) was added MeMgCl (3.0 M in THF, 110 μL, 0.33 mmol) at room temperature and the resultant yellow mixture was stirred at room temperature for 1 h. After a small amount of 1,4-dioxane was added, the mixture was stirred at room temperature for 5 min and the solvent was removed *in vacuo*. After the addition of hexane (6 mL) to the yellow residue, the solution was filtered through Celite, and the filter cake was washed with hexane (2 mL, 4 times). The combined filtrate was concentrated to *ca.* 6 mL and the solution was kept at −30 ˚C to give **4** as yellow crystals, which were collected by decantation and dried *in vacuo* (110 mg, 0.243 mmol, 81%). ^{1}H NMR (C_6D_6) δ −6.6, −15.8, −20.3. Magnetic susceptibility (Evans' method): μ_{eff} = 3.6±0.2 μ_B in C_6D_6 at 296 K. Anal. Calcd. for $C_{23}H_{45}FeNP_2$: C, 60.93; H, 10.00; N, 3.09. Found: C, 61.16; H, 10.00; N, 3.27.

Preparation of 5.

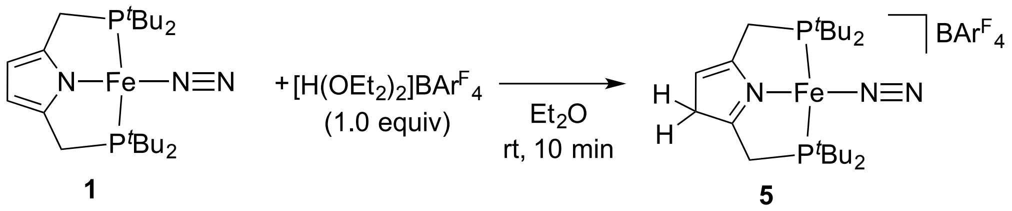

To a red solution of **1** (9.7 mg, 0.021 mmol) in Et_2O (1 mL) was added a colorless solution of $[H(OEt_2)_2]BAr^F_4$ (21.5 mg, 0.021 mmol) in Et_2O (1.5 mL) at room temperature and the resultant blue mixture was stirred at room temperature for 10 min under N_2 (1 atm). No formation of dihydrogen was confirmed by a GC. The mixture was filtered and the filter cake was washed with Et_2O (0.5 mL, 3 times). Slow addition of pentane (4 mL) to the concentrated filtrate (*ca.* 1 mL) at −17 ˚C afforded **5** as a blue crystalline solid, which was collected by decantation and dried *in vacuo* (25.8 mg, 0.019 mmol, 90%). No peak was observed in $^{31}P\{^1H\}$ NMR (Et_2O) of **5**. Further NMR analysis could not be carried out because **5** was unstable in solvents other than Et_2O. IR (KBr, cm^{-1}) 2034 (ν_{NN}). IR (Et_2O, cm^{-1}) 2026 (ν_{NN}). Anal. Calcd. for $C_{54}H_{55}BF_{24}FeN_3P_2$: C, 48.74; H, 4.17; N, 3.16. Found: C, 48.52; H, 3.87; N, 3.11.

Stoichiometric Reaction of 3 with $[H(OEt_2)_2]BAr^F_4$ and KC_8 under N_2.

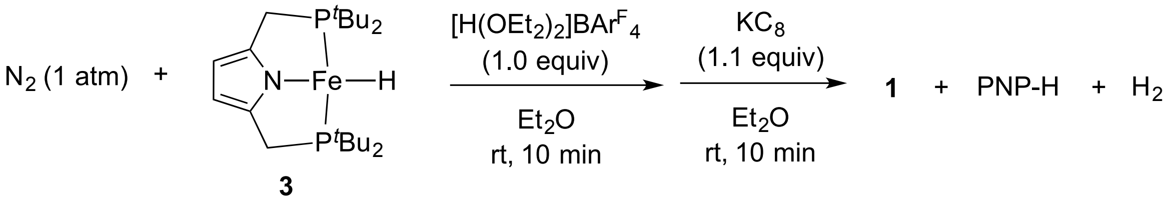

To a mixture of **3** (8.6 mg, 0.020 mmol) and $[H(OEt_2)_2]BAr^F_4$ (20.2 mg, 0.020 mmol) was added Et_2O (1 mL) under N_2 (1 atm) and the mixture was stirred at room temperature for 10 min. The evolved gas was analyzed by a GC and H_2 (0.006 mmol, 30% yield based on **3**) was detected. To the mixture was added a suspension of KC_8 (3.0 mg, 0.022 mmol) in Et_2O (1 mL) and the mixture was stirred at room temperature for 10 min. The solvent was removed *in vacuo.* After the addition of hexane (3 mL) to the residue, the solution was filtered through Celite, and the filter cake was washed with hexane (1 mL, 3 times). The combined filtrate was evaporated and dried *in vacuo* to afford a red solid. IR spectrum of the solid in KBr showed a strong absorption at 1967

cm^{-1}, which was identical to ν_{NN} band of the dinitrogen complex **1**. The solid was dissolved in C_6D_6 containing naphthalene as an internal standard and the integration of ^1H NMR resonances revealed the formation of **1** in 29% yield, PNP-H in 24% yield, and the recovery of **3** in 4%.

Stoichiometric Reaction of 4 with $[H(OEt_2)_2]BAr^F_4$ and KC_8 under N_2.

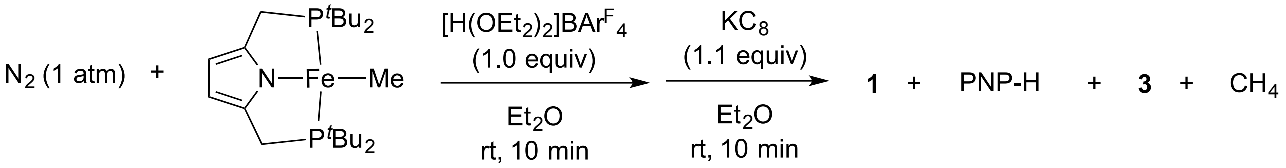

4

To a mixture of **4** (8.9 mg, 0.020 mmol) and $[H(OEt_2)_2]BAr^F_4$ (20.6 mg, 0.020 mmol) was added Et_2O (1 mL) under N_2 (1 atm) and the mixture was stirred at room temperature for 10 min. The evolved gas was analyzed by a GC and CH_4 (0.008 mmol, 40% yield based on **4**) was detected. To the mixture was added a suspension of KC_8 (3.0 mg, 0.022 mmol) in Et_2O (1 mL) and the mixture was stirred at room temperature for 10 min. The solvent was removed *in vacuo*. After the addition of hexane (3 mL) to the residue, the solution was filtered through Celite, and the filter cake was washed with hexane (1 mL, 3 times). The combined filtrate was evaporated and dried *in vacuo* to afford an orange solid. IR spectrum of the solid in KBr showed a strong absorption at 1967 cm^{-1}, which was identical to ν_{NN} band of the dinitrogen complex **1**. The solid was dissolved in C_6D_6 containing naphthalene as an internal standard and the integration of ^1H NMR resonances revealed the formation of **1** in 17% yield, PNP-H in 21% yield, **3** in 3% yield, and the recovery of **4** in 36%.

Reaction of 5 with KC_8.

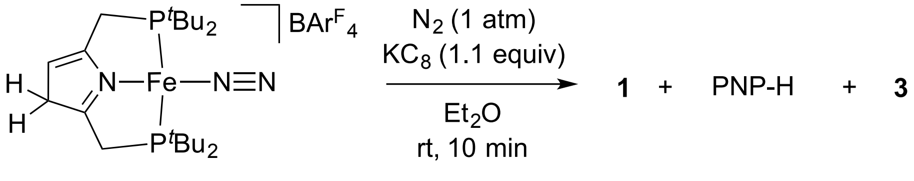

To a mixture of **5** (19.0 mg, 0.014 mmol) and KC_8 (2.3 mg, 0.017 mmol) was added Et_2O (1 mL) under N_2 (1 atm) and the resultant red mixture was stirred at room temperature for 10 min. After hexane (3 mL) was added to the mixture, the solution was filtered through Celite, and the

filter cake was washed with hexane (1 mL, 3 times). The combined filtrate was evaporated and dried *in vacuo* to afford a red solid. IR spectrum of the solid in KBr showed a strong absorption at 1963 cm^{-1}, which was identical to ν_{NN} band of the dinitrogen complex **1**. The solid was dissolved in C_6D_6 containing naphthalene as an internal standard and the integration of ^1H NMR resonances revealed the formation of **1** in 38% yield, PNP-H in 27% yield and **3** in 3% yield.

Reduction of 2 with Excess Amount of Na in the Presence of 15-Crown-5 under N_2 (1 atm).

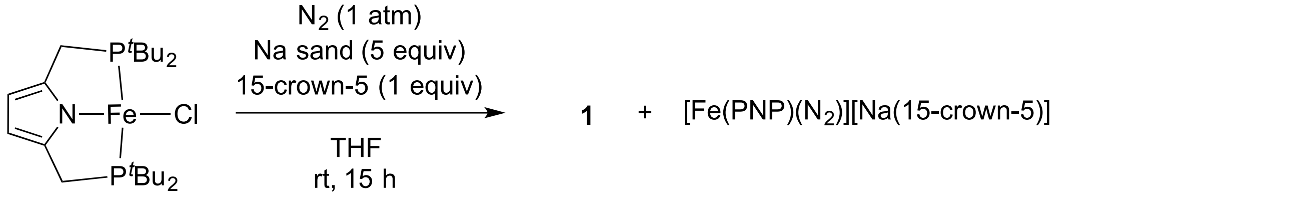

2

A suspension of **2** (28.1 mg, 0.059 mmol), Na sand (6.9 mg, 0.30 mmol), and 15-crown-5 (12 μL, 0.060 mmol) in THF (3 mL) was stirred at room temperature for 15 h under N_2 (1 atm). The resultant dark brownish purple suspension was filtered through Celite, and the filter cake was washed with THF (1.5 mL, 3 times). The combined filtrate was concentrated *in vacuo* to afford a brown solid. IR spectrum of the solid in KBr showed a strong absorption at 1966 cm^{-1}, which was identical to ν_{NN} band of the dinitrogen complex **1**. The brown solid was washed with hexane (1 mL, 3 times) and dried *in vacuo*. Recrystallization from THF-hexane (2 mL/3 mL) afforded a small amount of brown crystals. In the IR spectrum in a solid state (KBr), these brown crystals showed ν_{NN} peaks at 1831 cm-1. The preliminary result of X-ray analysis indicates the formation of an iron-dinitrogen complex [Fe(PNP)(N_2)][Na(15-crown-5)]. During the operation of the isolation of this complex, the decomposition of the complex was observed.

X-ray Crystallography.

Crystallographic data of **1**-**4** are summarized in Supplementary Tables 1 and 2. Selected bond lengths, angles and their ORTEP drawings are shown in Supplementary Fig. 3–6. Diffraction data for **1**-**4** were collected for the 2θ range of 5° to 55° at −150 °C on a Rigaku RAXIS RAPID imaging plate area detector with graphite-monochromated Mo Kα radiation (λ = 0.71075 Å), with VariMax optics. Intensity data were collected for Lorenz-polarization effects and for empirical absorption (REQAB). The structure solution and refinements were carried out by using the *CrystalStructure* crystallographic software package.[11] The positions of the non-hydrogen atoms were determined by direct methods (SIR 97[12] for **1**, **2**, and **3**, SIR2002[13] for **4**) and subsequent Fourier syntheses (DIRDIF-99[14]) and were refined F_o^2 using all unique reflections by full-matrix least-squares with anisotropic thermal parameters. All the other hydrogen atoms were placed at the calculated positions with fixed isotropic parameter, except for the H(43) atom in **3**. The position of the H(43) atom in **3** was determined on peaks in the difference Fourier maps and further refined isotropically.

Catalytic Reduction of Dinitrogen to Ammonia and Hydrazine under N_2 (1 atm).

$$\underset{(1\ \text{atm})}{N_2} + \underset{(40\ \text{equiv})}{KC_8} + \underset{(38\ \text{equiv})}{[H(OEt_2)_2]BAr^F_4} \xrightarrow[\substack{Et_2O \\ -78\ ^\circ C,\ 1\ h}]{\substack{\text{cat.} \\ (0.010\ \text{mmol})}} NH_3 + NH_2NH_2 + H_2$$

A catalytic reduction of dinitrogen into ammonia and hydrazine was carried out according to a method similar to the Peters' procedure.[15-17] A typical experimental procedure using **1** is described below.

In a 50 mL Schlenk flask were placed **1** (4.6 mg, 0.010 mmol), KC_8 (54.2 mg, 0.401 mmol), and $[H(OEt_2)_2]BAr^F_4$ (385 mg, 0.380 mmol). After the mixture was cooled to −196 ˚C, Et_2O (5 mL) was added to the mixture by trap-to-trap distillation. The Schlenk flask was warmed to −78 ˚C and then was filled with N_2 (1 atm). After stirring at −78 ˚C for 1 h, the mixture was warmed to room temperature and further stirred at room temperature for 20 min. The amount of dihydrogen of the catalytic reaction was determined by GC analysis. The reaction mixture was evaporated under reduced pressure, and the distillate was trapped in dilute H_2SO_4 solution (0.5 M, 10 mL). Aqueous solution of potassium hydroxide (30 wt%, 5 mL) was added to the residue, and the mixture was distilled into another dilute H_2SO_4 solution (0.5 M, 10 mL). The amount of NH_3 present in each of the H_2SO_4 solutions was determined by the indophenol method.[18] The amount of NH_2NH_2 present in each of the H_2SO_4 solutions was determined by the *p*-(dimethylamino)benzaldehyde method.[19]

Typical results of investigation on catalytic reduction of dinitrogen to ammonia and hydrazine under various conditions are summarized in Supplementary Tables 3-5.

Catalytic Reduction of Dinitrogen in Et_2O under $^{15}N_2$.

$$\underset{\text{(1 atm)}}{^{15}N_2} + \underset{\text{(40 equiv)}}{6\ KC_8} + \underset{\text{(38 equiv)}}{6\ [H(OEt_2)_2]BAr^F{}_4} \xrightarrow[\substack{Et_2O \\ -78\ ^\circ C,\ 1\ h}]{\substack{\text{cat. } \mathbf{4} \\ (0.010\ \text{mmol})}} \xrightarrow{HCl} 2\ [^{15}NH_4]Cl$$

In a 50 mL Schlenk flask were placed **4** (4.6 mg, 0.010 mmol), KC_8 (53.9 mg, 0.399 mmol), $[H(OEt_2)_2]BAr^F{}_4$ (385 mg, 0.380 mmol). After the mixture was cooled to −196 ˚C, Et_2O (5 mL) was added to the mixture by trap-to-trap distillation. The Schlenk flask was filled with $^{15}N_2$ (1 atm) and then was warmed to −78 ˚C. After stirring at −78 ˚C for 1 h, the mixture was warmed to room temperature and further stirred at room temperature for 20 min. After the catalytic reduction was completed, the mixture was cooled to −78 ˚C. To the mixture was added a solution of KO^tBu (2 mmol) in THF−MeOH (1 mL/2 mL) at −78 ˚C and the mixture was stirred at room temperature for 30 min. The volatile components in the mixture were collected to another Schlenk flask. To the obtained volatiles was added HCl in Et_2O (2 M, 2.5 mL, 5 mmol). The solvent was dried *in vacuo* to afford a solid which contains $^{15}NH_4Cl$ (0.023 mmol, 2.3 equiv). The amount of $^{15}NH_4Cl$ was determined by ^1H NMR using 1,1,2,2-tetrachloroethane as an internal standard. ^1H NMR (DMSO-d_6) δ 7.31 (d, $J_{N\text{-}H}$ = 71.3 Hz, $^{15}NH_4Cl$). $^{15}N\{^1H\}$ NMR (DMSO-d_6) δ −353.8 (s, $^{15}NH_4Cl$). The $^{15}N\{^1H\}$ NMR spectrum was shown in Supplementary Fig. 8.

Catalytic Reduction of Dinitrogen in THF under $^{15}N_2$.

$$\underset{(1\ \text{atm})}{^{15}N_2} + \underset{(40\ \text{equiv})}{KC_8} + \underset{(38\ \text{equiv})}{[H(OEt_2)_2]BAr^F{}_4} \xrightarrow[\substack{\text{THF} \\ -78\ ^\circ\text{C, 1 h}}]{\substack{\text{cat. } \mathbf{1} \\ (0.010\ \text{mmol})}} \xrightarrow{\text{HCl}} [^{15}NH_4]Cl + [^{15}NH_2{}^{15}NH_3]Cl$$

In a 50 mL Schlenk flask were placed **1** (4.6 mg, 0.010 mmol), KC_8 (54.5 mg, 0.403 mmol), $[H(OEt_2)_2]BAr^F{}_4$ (385 mg, 0.380 mmol). After the mixture was cooled to −196 ˚C, THF (5 mL) was added to the mixture by trap-to-trap distillation. The Schlenk flask was filled with $^{15}N_2$ (1 atm) and then was warmed to −78 ˚C. After stirring for at −78 ˚C 1 h, the mixture was warmed to room temperature and further stirred at room temperature for 20 min. After the catalytic reduction was completed, the mixture was cooled to −78 ˚C. To the mixture was added a solution of KO^tBu (2 mmol) in THF−MeOH (1 mL/2 mL) at −78 ˚C and the mixture was stirred at room temperature for 30 min. The volatile components in the mixture were collected to another Schlenk flask. To the obtained volatiles was added HCl in Et_2O (2 M, 2.5 mL, 5 mmol). The solvent was dried *in vacuo* to afford a solid which contains $^{15}NH_4Cl$ (0.020 mmol, 2.0 equiv) and $^{15}NH_2{}^{15}NH_3Cl$ (0.016 mmol, 1.6 equiv). The amounts of $^{15}NH_4Cl$ and $^{15}NH_2{}^{15}NH_3Cl$ were determined by ^1H NMR using 1,1,2,2-tetrachloroethane as an internal standard. ^1H NMR (DMSO-d_6) δ 7.30 (d, $J_{N\text{-}H}$ = 71.0 Hz, $^{15}NH_4Cl$), 6.35 (br s, $^{15}NH_2{}^{15}NH_3Cl$). $^{15}N\{^1H\}$ NMR (DMSO-d_6) δ −328.4 (s, $^{15}NH_2{}^{15}NH_3Cl$),[20] −353.9 (s, $^{15}NH_4Cl$). The $^{15}N\{^1H\}$ NMR spectrum was shown in Supplementary Fig. 9.

Reaction of 1 with 20 Equiv of KC_8 and 19 Equiv of $[H(OEt_2)_2]BAr^F{}_4$ under N_2 Atmosphere.

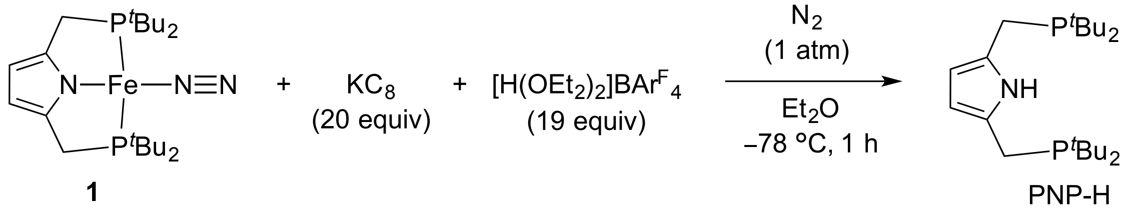

In a 50 mL Schlenk flask were placed **1** (9.2 mg, 0.020 mmol), KC_8 (54.3 mg, 0.402 mmol), $[H(OEt_2)_2]BAr^F{}_4$ (384 mg, 0.379 mmol). After the mixture was cooled at −196 ˚C, Et_2O (5 mL) was added to the mixture by trap-to-trap distillation. The Schlenk flask was warmed to −78 ˚C and then was filled with N_2 (1 atm). After stirring at −78 ˚C for 1 h, the mixture was warmed to room temperature and further stirred at room temperature for 40 min. The solvent was removed *in vacuo*. To the residue was added hexane (5 mL), the suspension was filtered through Celite, and the filter cake was washed with hexane (3 mL, 2 times). The combined filtrate was evaporated and dried *in vacuo* to afford a colorless oil. The residue was dissolved in C_6D_6 containing naphthalene as an internal standard and the integration of ^1H NMR resonances revealed the formation of PNP-H in 48% yield.

Reaction of Hydrazine with 1, KC_8, and $[H(OEt_2)_2]BAr^F_4$ under N_2 Atmosphere.

$$\underset{\text{(1 atm)}}{N_2} + \underset{\text{(2 equiv)}}{NH_2NH_2} + \underset{\text{(40 equiv)}}{KC_8} + \underset{\text{(38 equiv)}}{[H(OEt_2)_2]BAr^F_4} \xrightarrow[\substack{Et_2O \\ -78\ ^\circ C,\ 1\ h}]{\substack{\text{cat. } \mathbf{1} \\ (0.010\ mmol)}} NH_3 + NH_2NH_2 + H_2$$

In a 50 mL Schlenk flask were placed **1** (4.6 mg, 0.010 mmol), KC_8 (54.2 mg, 0.401 mmol), $[H(OEt_2)_2]BAr^F_4$ (385 mg, 0.380 mmol), and After the mixture was cooled at −196 ˚C, Et_2O (5 mL) and anhydrous hydrazine (0.6 μl, 0.020 mmol) were added to the mixture by trap-to-trap distillation. The Schlenk flask was warmed to −78 ˚C and then was filled with N_2 (1 atm). After stirring at −78 ˚C for 1 h, the mixture was warmed to room temperature and further stirred at room temperature for 20 min. The amount of dihydrogen (1.2 equiv) was determined by GC analysis. The reaction mixture was evaporated under reduced pressure, and the distillate was trapped in dilute H_2SO_4 solution (0.5 M, 10 mL). Aqueous solution of potassium hydroxide (30 wt%, 5 mL) was added to the residue, and the mixture was distilled into another dilute H_2SO_4 solution (0.5 M, 10 mL). The amount of NH_3 present in each of the H_2SO_4 solutions was determined by the indophenol method.[18] The amount of NH_2NH_2 present in each of the H_2SO_4 solutions was determined by the *p*-(dimethylamino)benzaldehyde method.[19] The obtained amounts of ammonia and hydrazine were 5.0 equiv and 1.4 equiv, respectively.

Reaction of Hydrazine with Iron Complexes, KC_8, and $[H(OEt_2)_2]BAr^F_4$ under Argon Atmosphere.

A typical experimental procedure is described below. In a 50 mL Schlenk flask were placed catalyst (0.010 mmol), KC_8, and $[H(OEt_2)_2]BAr^F_4$. After the mixture was cooled at −196 ˚C, Et_2O (5 mL) and anhydrous hydrazine (1.3 μl, 0.040 mmol) were added to the mixture by trap-to-trap distillation. The Schlenk flask was warmed to −78 ˚C and then was filled with Ar (1 atm). After stirring at −78 ˚C for 1 h, the mixture was warmed to room temperature and further stirred at room temperature for 20 min. The amount of dihydrogen was determined by GC analysis. The reaction mixture was evaporated under reduced pressure, and the distillate was trapped in dilute H_2SO_4 solution (0.5 M, 10 mL). Aqueous solution of Potassium hydroxide (30 wt%, 5 mL) was added to the residue, and the mixture was distilled into another dilute H_2SO_4 solution (0.5 M, 10 mL). The amount of NH_3 present in each of the H_2SO_4 solutions was determined by the indophenol method.[18] The amount of NH_2NH_2 present in each of the H_2SO_4 solutions was determined by the *p*-(dimethylamino)benzaldehyde method.[19]

Typical results of investigation on the reaction of hydrazine under various conditions are summarized in Supplementary Table 6.

Catalytic Reduction of Dinitrogen into $N(SiMe_3)_3$.

$$\underset{(1\ \text{atm})}{N_2} + \underset{(600\ \text{equiv})}{6\ \text{Na}} + \underset{(600\ \text{equiv})}{6\ Me_3SiCl} \xrightarrow[\text{THF, rt, 20 h}]{\text{cat. (0.020 mmol)}} 2\ N(SiMe_3)_3 \xrightarrow[\text{rt}]{H_2SO_4} 2\ NH_3$$

A typical procedure for catalytic reduction of dinitrogen into $N(SiMe_3)_3$ using **1** is described below. To a suspension of sodium (277 mg, 12.0 mmol) and **1** (9.3 mg, 0.020 mmol) in THF (8 mL) was added Me_3SiCl (1.50 mL, 12.0 mmol) under N_2 (1 atm). The mixture was stirred at toom temperature for 20 h under a gentle steam of N_2 (1 atm). To the reaction mixture was added cyclododecane (84.6 mg) as an internal standard for gas-liquid chromatography (GLC) analysis. After stirring for 5 min, the mixrture was centrifuged to remove insoluble materials. The resultant clear supernatant was subjected to GLC and capillary gas chromatography mass spectrometry analyses. Then the supernatant was stirred with an aqueous solution of H_2SO_4 (0.5 M, 10 mL) for 12 h. After aqueous solution of potassium hydroxide (30 wt%, 5 mL) was added to the mixture, the mixture was distilled into H_2SO_4 solution (0.5 M, 10 mL). The amount of NH_3 present in the H_2SO_4 solutions was determined by the indophenol method.[18]

Typical results of investigation on the catalytic reduction of dinitrogen into $N(SiMe_3)_3$ under various conditions are shown in Supplementary Tables 7 and 8.

Computational Methods

DFT calculations were performed with the Gaussian 09 program (Rev. D.01).[21] Geometry optimizations were carried out with the B3LYP-D3 functional, which is the B3LYP hybrid functional[22-26] combined with an empirical dispersion correction developed by Grimme.[27] The SDD (Stuttgart/Dresden pseudopotentials) basis set[28,29] and 6-31G(d) basis sets[30-33] were employed for Fe atom and the other atoms, respectively. In the optimization calculations for the protonation processes, solvation effects of diethylether were taken into account by using the polarizable continuum model (PCM).[34] Optimized structures were confirmed to have the appropriate number of imaginary frequencies by vibrational analysis. Calculated vibrational frequencies were corrected with a scaling factor of 0.960.[35] An appropriate connection between a reactant and a product for each reaction step was confirmed by IRC[36,37] and quasi-IRC calculations. In the quasi-IRC calculation, the geometry of a transition state was at first shifted by perturbing the geometries very slightly along the reaction coordinate and released for equilibrium optimization. To discuss the energetics of the protonation processes, single-point energy calculations at the optimized geometries were performed at the B3LYP-D3/def2-TZVP[38,39] level of theory. Energy profiles are described by using the Gibbs free energy changes (ΔG) at 195 K.

Assessment of the appropriate functional

To determine the appropriate functional for the calculation of the present iron system, we have optimized the structure of **1** in the doublet and quartet spin states with DFT calculations using the B3LYP, B3LYP-D3, and M06[40] functionals. Selected geometric parameters of **1** are shown in Supplementary Table 9. Optimized structures of the doublet state of **1** do not depend on the choice of functionals. All the optimized structures are close to the X-ray crystal structure. Calculated relative energies of the doublet and quartet spin states of **1** are summarized in Supplementary Table 10. The M06 result predicts that the quartet state of **1** is 1.2 kcal/mol lies above the doublet state at the M06 level, while the B3LYP and B3LYP-D3 results are in consistent with the experimental result that the ground spin state of **1** at room temperature is doublet. In the present study, we adopted the B3LYP-D3 functional to ensure the correct description of nonbonding interactions in the protonation processes.

Supplementary References

1. Evans, D. F. Determination of the paramagnetic susceptibility of substances in solution by nuclear magnetic resonance. *J. Chem. Soc.* 2003-2004 (1959).

2. Live, D. H. & Chan, S. I. Bulk susceptibility corrections in nuclear magnetic resonance experiments using superconducting solenoids. *Anal. Chem.* **42**, 791-792 (1970).

3. Bain, G. A. & Berry, J. F. Diamagnetic corrections and Pascal's constants. *J. Chem. Educ.* **85**, 532-536 (2008).

4. Venkanna, G. T., Arman, H. D. & Tonzetich, Z. J. Catalytic C−S cross-coupling reactions employing Ni complexes of pyrrole-based pincer ligands. *ACS Catal.* **4**, 2941-2950 (2014).

5. Kreye, M., Freytag, M., Jones, P. G., Williard, P. G., Bernskoetter, W. H. & Walter, M. D. Homolytic H_2 cleavage by a mercury-bridged Ni(I) pincer complex [{(PNP)Ni}$_2${μ-Hg}]. *Chem. Commun.* **51**, 2946-2949 (2015).

6. Levine, D. S., Tilley, T. D. & Anderson, R. A. C−H bond activations by monoanionic, PNP-supported scandium dialkyl complexes. *Organometallics* **34**, 4647-4655 (2015).

7. Kern, R. J. *J. Inorg. Nucl. Chem.* Tetrahydrofuran complexes of transition metal chlorides. *J. Inorg. Nucl. Chem.* **24,** 1105-1109 (1962).

8. Wietz, I. S. & Rabinovitz, M. The application of C_8K for organic synthesis: reduction of substituted naphthalenes. *J. Chem. Soc., Perkin Trans.* **1**, 117-120 (1993).

9. Brookhart, M., Grant, B. & Volpe Jr., A. F. $[(3,5\text{-}(CF_3)_2C_6H_3)_4B]^-[H(OEt_2)_2]^+$: a convenient reagent for generation and stabilization of cationic, highly electrophilic organometallic complexes. *Organometallics* **11**, 3920-3922 (1992).

10. Robbins, J. L., Edelstein, N., Spencer, B. & Smart, J. C. Syntheses and electronic-structures of decamethylmetallocenes. *J. Am. Chem. Soc.* **104**, 1882-1893 (1982).

11. *CrystalStructure 4.0: Single Crystal Structure Analysis Software*; Rigaku Corp: Tokyo, Japan, and MSC: The Woodlands, TX, 2010.

12. *SIR-97*: Altomare, A.; Burla, M. C.; Camalli, M.; Cascarano, G. L.; Giacovazzo, C.; Guagliardi, A.; Moliterni, A. G. G.; Polidori, G.; Spagna, R. *J. Appl. Crystallogr.* **1999**, *32*, 115.

13. *SIR-2002*: Burla, M. C.; Camalli, M.; Carrozzini, B.; Cascarano, G. L.; Giacovazzo, C.; Polidori, G.; Spagna, R. *J. Appl. Crystallogr.* **2003**, *36*, 1103.

14. Beurskens, P. T., Beurskens, G., de Gelder, R., García-Granda, S., Gould, R. O., Israël, R. & Smits, J. M. M. *The DIRDIF-99 Program System*; Crystallography Laboratory, University of Nijmegen: Nijmegen, The Netherlands, 1999.

15. Anderson, J. S., Rittle, J. & Peters, J. C. Catalytic conversion of nitrogen to ammonia by an iron model complex. *Nature* **501**, 84-88 (2013).

16. Creutz, S. E. & Peters, J. C. Catalytic reduction of N_2 to NH_3 by an $Fe{-}N_2$ complex featuring a C-atom anchor. *J. Am. Chem. Soc.* **136**, 1105-1115 (2014).

17. Ung, G & Peters, J. C. Low-temperature N_2 binding to two-coordinate L_2Fe^0 enables reductive trapping of $L_2FeN_2^-$ and NH_3 generation. *Angew. Chem. Int. Ed.* **54**, 532-535 (2015).

18. Weatherburn, M. W. Phenol-hypochlorite reaction for determination of ammonia. *Anal. Chem.* **39**, 971 (1967).

19. Watt, G. W. & Chrisp, J. D. A spectrophotometric method for the determination of hydrazine. *Anal. Chem.* **24**, 2006 (1952).

20. Mock, M. T., Chen, S., O'Hagan, M., Rousseau, R., Dougherty, W. G., Kassel, W. S. & Bullock, R. M. Dinitrogen reduction by a chromium(0) complex supported by a 16-membered phosphorus macrocycle. *J. Am. Chem. Soc.* **135**, 11493-11496 (2013).

21. Frisch, M. J., Trucks, G. W., Schlegel, H. B., Scuseria, G. E., Robb, M. A., Cheeseman, J. R., Scalmani, G., Barone, V., Mennucci, B., Petersson, G. A., Nakatsuji, H., Caricato, M., Li, X., Hratchian, H. P., Izmaylov, A. F., Bloino, J., Zheng, G., Sonnenberg, J. L., Hada, M., Ehara, M., Toyota, K., Fukuda, R., Hasegawa, J., Ishida, M., Nakajima, T., Honda, Y., Kitao, O., Nakai, H., Vreven, T., Montgomery, J. A., Jr., Peralta, J. E., Ogliaro, F., Bearpark, M., Heyd, J. J., Brothers, E., Kudin, K. N., Staroverov, V. N., Keith, T., Kobayashi, R., Normand, J., Raghavachari, K., Rendell, A., Burant, J. C., Iyengar, S. S., Tomasi, J., Cossi, M., Rega, N., Millam, N. J., Klene, M., Knox, J. E., Cross, J. B., Bakken, V., Adamo, C., Jaramillo, J., Gomperts, R., Stratmann, R. E., Yazyev, O., Austin, A. J., Cammi, R., Pomelli, C., Ochterski, J. W., Martin, R. L., Morokuma, K., Zakrzewski, V. G., Voth, G. A., Salvador, P., Dannenberg, J. J., Dapprich, S., Daniels, A. D., Farkas, O., Foresman, J. B., Ortiz, J. V., Cioslowski, J. & Fox, D. J. *Gaussian 09*, Revision C.01; Gaussian, Inc.: Wallingford CT, 2010.

22. Becke, A. D. Density-functional exchange-energy approximation with correct asymptotic

behavior. *Phys. Rev.* A **38**, 3098-3100 (1988).

23. Becke, A. D. Density-functional thermochemistry. III. The role of exact exchange. *J. Chem. Phys.* **98**, 5648-5652 (1993).

24. Lee, C., Yang, W. & Parr, R. G. Development of the Colle-Salvetti correlation-energy formula into a functional of the electron density. *Phys. Rev. B* **37**, 785-789 (1988).

25. Vosko, S. H., Wilk, L. & Nusair, M. J. Accurate spin-dependent electron liquid correlation energies for local spin density calculations: A critical analysis. *Can. J. Phys.* **58**, 1200–1211 (1980).

26. Lee, C., Yang, W. & Parr, R. G. Development of the Colle-Salvetti correlation energy formula into a functional of the electron density. *Phys. Rev. B* **37**, 785–789 (1988).

27. Grimme, S., Antony, J., Ehrlich, S. & Krieg, H. A consistent and accurate *ab initio* parameterization of density functional dispersion correction (DFT-D) for the 94 elements H-Pu. *J. Phys. Chem.* **132**, 154104 (2010).

28. Dolg, M., Wedig, U., Stoll, H. & Preuß, H. Energy-adjusted *ab initio* pseudopotentials for the first row transition elements. *J. Chem. Phys.* **86**, 866-872 (1987).

29. Andrae, D., Häußermann, U., Dolg, M., Stoll, H. & Preuß, H. Energy-adjusted *ab initio* pseudopotentials for the second and third row transition elements. *Theor. Chim. Acta.* **77**, 123-141 (1990).

30. Ditchfield, R., Hehre, W. J. & Pople, J. A. Self-consistent molecular-orbital methods. IX. An extended Gaussian-type basis for molecular-orbital studies of organic molecules. *J. Chem. Phys.* **54**, 724-728 (1971).

31. Hehre, W. J., Ditchfield, R. & Pople, J. A. Self-consistent molecular orbital methods. XII. Further extensions of Gaussian-type basis sets for use in molecular orbital studies of organic molecules. *J. Chem. Phys.* **56**, 2257-2261 (1972).

32. Hariharan, P. C. & Pople, J. A. The influence of polarization functions on molecular orbital hydrogenation energies. *Theor. Chem. Acc.* **28**, 213-222 (1973).

33. Francl, M. M., Pietro, W. J., Hehre, W. J., Binkley, J. S., Gordon, M. S., DeFrees, D. J. & Pople, J. A. Self-consistent molecular orbital methods. XXIII. A polarization-type basis set for second-row elements. *J. Chem. Phys.* **77**, 3654-3665 (1982).

34. Tomasi, J., Mennucci, B. & Cammi, R. Quantum mechanical continuum solvation models.

Chem. Rev. **105**, 2999-3094 (2005).

35. Scott, A. P. & Radom, L. Harmonic vibrational frequencies: an evaluation of Hartree-Fock, Møller-Plesset, quadratic configuration interaction, density functional theory, and semiempirical scale factors. *J. Phys. Chem.* **100**, 16502-16513 (1996).

36. Fukui, K. Formulation of the reaction coordinate. *J. Phys. Chem.* **74**, 4161-4163 (1970).

37. Fukui, K. The path of chemical reactions–the IRC approach. *Acc. Chem. Res.* **14**, 363-368 (1981).

38. Weigend, F. & Ahlrichs, R. Balanced basis sets of split valence, triple zeta valence and quadruple zeta valence quality for H to Rn: Design and assessment of accuracy. *Phys. Chem. Chem. Phys.* **7**, 3297-3305 (2005).

39. Weigend, F. Accurate Coulomb-fitting basis sets for H to Rn. *Phys. Chem. Chem. Phys.* **8**, 1057-1065 (2006).

40. Zhao, Y. & Truhlar, D. G. The M06 suite of density functionals for main group thermochemistry, thermochemical kinetics, noncovalent interactions, excited states, and transition elements: two new functionals and systematic testing of four M06-class functionals and 12 other functionals. *Theor. Chem. Acc.* **120**, 215-241 (2008).
